# The bone marrow of mouse-rat chimeras contains progenitors of multiple pulmonary cell lineages

**DOI:** 10.3389/fcell.2024.1394098

**Published:** 2024-04-17

**Authors:** Enhong Li, Bingqiang Wen, Dengfeng Gao, Timothy R. Kalin, Guolun Wang, Tanya V. Kalin, Vladimir V. Kalinichenko

**Affiliations:** ^1^ Phoenix Children’s Research Institute, Department of Child Health, University of Arizona College of Medicine-Phoenix, Phoenix, AZ, United States; ^2^ State Key Laboratory of Animal Biotech Breeding, College of Biological Sciences, China Agricultural University, Beijing, China; ^3^ College of Arts and Sciences, University of Cincinnati, Cincinnati, OH, United States; ^4^ Division of Pulmonary Biology, Cincinnati Children’s Hospital Medical Center, Cincinnati, OH, United States; ^5^ Center for Cancer and Blood Diseases, Phoenix Children’s Hospital, Phoenix, AZ, United States; ^6^ Division of Neonatology, Phoenix Children’s Hospital, Phoenix, AZ, United States

**Keywords:** mouse-rat chimera, blastocyst injection, lung, bone marrow, respiratory progenitor cells

## Abstract

Radiation-induced lung injury (RILI) is a common complication of anti-cancer treatments for thoracic and hematologic malignancies. Bone marrow (BM) transplantation restores hematopoietic cell lineages in cancer patients. However, it is ineffective in improving lung repair after RILI due to the paucity of respiratory progenitors in BM transplants. In the present study, we used blastocyst injection to create mouse-rat chimeras, these are artificial animals in which BM is enriched with mouse-derived progenitor cells. FACS-sorted mouse BM cells from mouse-rat chimeras were transplanted into lethally irradiated syngeneic mice, and the contribution of donor cells to the lung tissue was examined using immunostaining and flow cytometry. Donor BM cells provided long-term contributions to all lung-resident hematopoietic cells which includes alveolar macrophages and dendritic cells. Surprisingly, donor BM cells also contributed up to 8% in pulmonary endothelial cells and stromal cells after RILI. To identify respiratory progenitors in donor BM, we performed single-cell RNA sequencing (scRNAseq). Compared to normal mouse BM, increased numbers of hematopoietic progenitors were found in the BM of mouse-rat chimeras. We also identified unique populations of hemangioblast-like progenitor cells expressing *Hes1*, *Dntt* and *Ebf1,* along with mesenchymal stromal cells expressing *Cpox*, *Blvrb* and *Ermap* that were absent or ultra-rare in the normal mouse BM. In summary, by using rats as “bioreactors”, we created a unique mouse BM cell transplant that contributes to multiple respiratory cell types after RILI. Interspecies chimeras have promise for future generations of BM transplants enriched in respiratory progenitor cells.

## Introduction

Radiation-induced lung injury (RILI) is caused by radiation exposure, a common side effect of anti-cancer therapies (Yan et al., 2022). Currently, treatment methods for RILI include symptomatic and supportive care that can include supplemental oxygen, infection control, and the use of anti-inflammatory medications (Hanania et al., 2019). Mesenchymal stromal cell (MSC) therapy is an emerging approach in the treatment of various lung diseases. MSCs are easily sourced, isolated, and expanded, making them increasingly recognized by healthcare professionals as a valuable source of cell therapies (Zhuang et al., 2021). Using human amnion-derived MSCs, the acute lung injury (ALI) in mice was alleviated by reducing endothelial permeability, oxidative stress, pro-inflammatory mediators, and lung tissue damage (Wu et al., 2023). Alveolar epithelial type II cells (AECIIs) differentiated *in vitro* from BM progenitor cells alleviated the inflammation and pathological lung tissue damage in a mouse model of ALI (Ma et al., 2023). In a rat ALI model, exogenously supplied BM progenitor cells reduced pulmonary epithelial permeability and improved lung repair after injury (Zhang et al., 2016). Intratracheal transplantation of human umbilical cord blood-derived MSCs resulted in long-term regenerative effects after hyperoxia-induced lung injury in neonatal mice with no long-term off-target complications (Ahn et al., 2013). Transplantation of MSCs in patients with pulmonary fibrosis enhanced their exercise capacity but did not reduce mortality (Averyanov et al., 2020). MSC transplantation in patients with acute myocardial infarction did not cause adverse reactions as determined by chest X-rays, lung function tests, and the lack of evidence for tumorigenic lesions or pulmonary fibrosis changes (Hare et al., 2009).

Hematopoietic stem cells (HSCs) reside in BM, self-renew and differentiate into multipotent progenitor cells (MPPs), initiating the process of hematopoiesis (Oguro et al., 2013). MPPs further differentiate into common myeloid progenitors (CMPs), lymphoid-primed multipotent progenitors (LMPPs), and common lymphoid progenitors (CLPs). As BM cell division progresses, lineage restrictions occur, ultimately leading to the generation of all mature blood cell types. While BM contains hemangioblast-like cells (HABs) which are bipotential progenitors for both blood and endothelial cell lineages (Mikkola and Orkin, 2006). BM-derived HABs are rare. Various BM-derived cell types circulate throughout the body in the peripheral blood and enter different organ tissues to perform their respective functions. Dendritic cell populations in radiation-injured lungs can be restored via bone marrow transplantation (Hahn et al., 2011). BM monocytes can differentiate into alveolar macrophages (AM) that require CD44 interaction with hyaluronan to promote AM cell survival (Dong et al., 2018). These results showed that both dendritic cells and alveolar macrophages in the lung tissue can derive from donor BM transplants. Published studies demonstrated that progenitor cells for lung endothelial cell (EC) lineages originate from both lung resident ECs and BM-derived circulating endothelial progenitor cells expressing CD31 and CD45 (Balasubramaniam et al., 2007). Bipotential HABs and hemogenic endothelium are common in embryonic tissues such as the yolk sac and dorsal aorta (Nishikawa et al., 1998). However, their presence in adult lung tissue has not been documented. It is unclear whether HABs can be isolated from bone marrow and used for lung regenerative medicine.

Using the blastocyst injection technology, differentiation of multiple cell lineages from pluripotent embryonic stem cells (ESCs) can be achieved in intraspecies (within a species) and interspecies (between species) chimeras. Blastocyst injection involves the transfer of donor ESCs into early embryos (blastocysts) of recipient animals to form chimeras. Donor ESCs undergo differentiation within recipient embryos that act as “biological reactors”, providing growth factors, hormones, and cellular microenvironments to promote and guide ESC differentiation in endogenous cell niches. Recently, we generated fully functional BM from mouse ESCs using mouse-rat interspecies chimeras (Wen et al., 2022). ESC-derived BM rescued lethally irradiated syngeneic mice and provided the long-term reconstitution to all hematopoietic cell lineages in the bone marrow and peripheral blood (Wen et al., 2022). The chimeric BM from mouse-rat chimeras was enriched in mouse-derived HSCs and other hematopoietic progenitor cells (Wen et al., 2022). While donor BM cells from mouse-rat chimeras exhibited increased regenerative capacity for the BM and peripheral blood of lethally irradiated recipient mice (Wen et al., 2022), it is unclear whether chimeric BM contains progenitor cells for non-hematopoietic cell lineages.

In this study, we used blastocyst injection of mouse ESCs and rat blastocysts to generate mouse-rat chimeras and examine the capacity of the chimeric ESC-derived BM cells to contribute to respiratory cell lineages in lethally irradiated syngeneic mice. ESC-derives donor BM cells differentiated into multiple respiratory cell lineages including lung-resident dendritic cells, alveolar macrophages, and cells of stromal, endothelial, and epithelial origins. Using single-cell RNA sequencing, we identified unique subsets of ESC-derived cells within the bone marrow of mouse-rat chimeras, such as hemangioblast-like and mesenchymal stromal cells, with distinct gene expression signatures compared to normal mouse BM. The present study highlights the potential of the interspecies chimeric technology to simultaneously produce multiple respiratory progenitors from pluripotent ESCs for lung regenerative medicine.

## Methods

Mice, rats and generation of mouse-rat and mouse-mouse chimeras through blastocyst injection. Interspecies mouse-rat chimeras were generated using blastocyst injection as described (Li et al., 2021; Wang et al., 2021). Briefly, blastocysts from SD rats were obtained at embryonic day 4.5 (E4.5), injected with fifteen GFP-labeled mouse ES cells (ESCs-GFP, C57BL/6 background) (Wen et al., 2021) and transferred into pseudo-pregnant SD rat females. Mouse-mouse chimeras were generated by complementing CD1 blastocysts with 15 mouse ESC-GFP cells. For FACS analysis and BM transplantation, BM cells were obtained from chimeric pups that were collected between postnatal day 4 (P4) and postnatal day 10 (P10). For single-cell RNA sequencing, BM cells were prepared from P10 chimeras. To perform BM cells transplantation, BM cells from two tibias and two fibulas of mouse-rat chimeras were FACS-sorted for ESC-derived (GFP^+^) cells. 500,000 of FACS-sorted GFP^+^ BM cells (pooled from five to nine mouse-rat chimeras) were intravenously (i.v.) injected into lethally radiated C57BL/6 male mice (6–8 weeks of age) via the tail vein. Three hours before BM cell transplantation, whole-body irradiation was performed using 11.75 Gy. Mice were harvested 8 days and 5 months after BM transplantation. Tissue dissection, processing and preparation of single-cell suspensions were carried out as described (Kalinichenko et al., 2003; Kim et al., 2005; Malin et al., 2007; Kalin et al., 2008; Bolte et al., 2011).

Single-Cell RNA sequencing analysis of ESC-derived bone marrow cells. Prior to single-cell RNA sequencing (scRNAseq) (10X Chromium platform), BM cells were pooled from three P10 mouse-rat chimeras and three P10 mouse-mouse (control) chimeras and then FACS-sorted for GFP and the *lineage* (Lin) marker. Since the numbers of HSCs and other progenitors in BM are significantly low compared to numbers of differentiated hematopoietic cells, the cell mixtures were enriched for BM progenitor cell populations by combining 90% of FACS-sorted GFP^+^Lin^–^cells and 10% of FACS-sorted GFP^+^Lin^+^ cells in each experimental group. This enrichment enabled us to obtain enough progenitor cells for UMAP clustering analysis. All raw data and the processed count matrix of BM cells datasets were uploaded to the GEO database (accession number GSE184940). Read alignments, quality controls and false discovery rates were described previously (Ren et al., 2019; Wang et al., 2022; Wen et al., 2024). Identification of cell clusters and quantification of cluster-specific gene expression in BM cells scRNAseq datasets was performed as described (Wen et al., 2022). To assess the transcriptomic similarity of ESC-derived and endogenous BM cells, the scRNAseq datasets were normalized using the *SCTransform* function and then integrated utilizing the canonical correlation analysis (CCA). In the integrated scRNAseq datasets, the *SelectIntegrationFeatures* function in Seurat package (version 4.0.0 in R 4.0 statistical environment) was used to identify anchors for integration. The *RunPCA* function was used for principal component analysis (PCA) of scRNAseq datasets, and the *PCElbowPlot* function was used to calculate the standard deviations of the principal components (PCs). PCs with standard deviation >3.5 were chosen as input parameters for non-linear UMAP clustering analysis. Next, the *FindNeighbors* function was used to compute the k. param nearest neighbors, and BM cells clusters were identified by a shared nearest neighbor (SNN) modularity optimization clustering algorithm implemented in the *FindClusters* function with resolution set at 0.4 (Wang et al., 2021; Wen et al., 2021).

FACS Analysis. FACS analysis was performed using cells obtained from the mouse lung. Primary antibodies for FACS analysis and staining conditions are listed in Supplementary Table SE3 (Bolte et al., 2017; Dunn et al., 2018; Shukla et al., 2019; Bian et al., 2023; Kolesnichenko et al., 2023; Pradhan et al., 2023). Immunostaining of cell suspensions was performed as described (Ren et al., 2010; Xia et al., 2015; Bolte et al., 2017). Identification of cell types based on multiple cell surface markers is described in (Pradhan et al., 2019; Bolte et al., 2020). Stained cells were analyzed using a five-laser FACSAria II (BD Biosciences) (Sun et al., 2021).

Histology and immunostaining. Frozen sections of tissue samples were imaged for GFP (Ustiyan et al., 2016; Ustiyan et al., 2018). Primary antibodies for immunostaining are listed in Supplementary Table SE3 (Kalinichenko et al., 2002; Lim et al., 2002; Wang et al., 2003; Ustiyan et al., 2012; Wang et al., 2012; Hoggatt et al., 2013). Secondary antibodies were conjugated with Alexa Fluor 488, Alexa Fluor 594 or Alexa Fluor 647 to visualize specific staining as described (Milewski et al., 2017a). DAPI (Vector Laboratory) was used to counterstain cell nuclei (Milewski et al., 2017b). Immunofluorescent images were obtained using a Zeiss Axioplan2 microscope (Carl Zeiss Microimaging) as described (Kalinichenko et al., 2001; Wang et al., 2010; Bolte et al., 2015).

Statistical Analysis. Statistical significance was determined using a non-parametric Mann-Whitney U test. *p* ≤ 0.05 was considered statistically significant. Data were presented as Mean ± Standard Deviation (SD).

## Results

**FIGURE 1:**
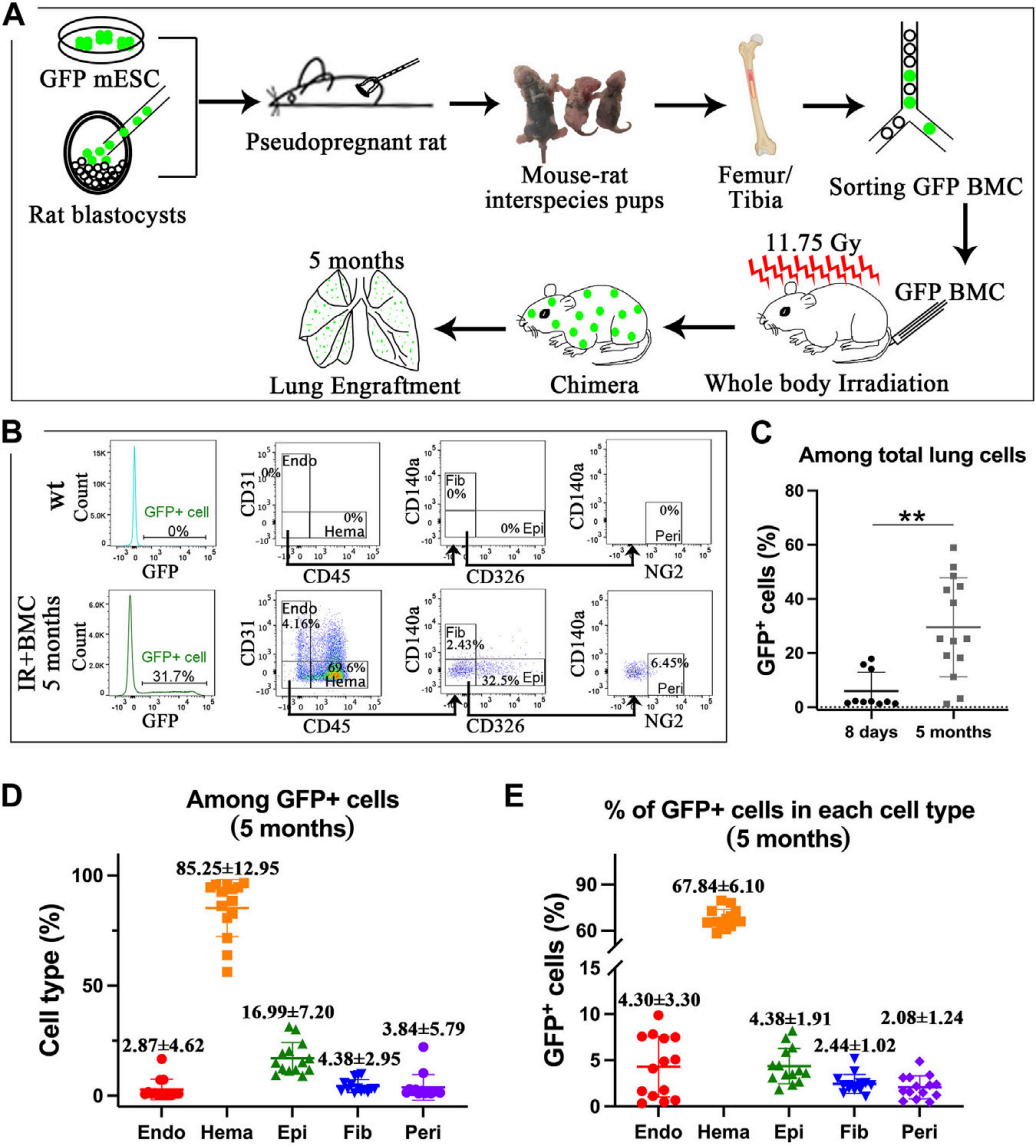
ESC-derived BM cells from mouse-rat chimeras contribute to lungs of lethally irradiated mice after BM transplantation. **(A)** Schematic diagram illustrating the rescue of lethally radiated mice by BM cells derived from GFP-labeled mouse ESCs. Mouse ESCs were injected into rat blastocysts, and the chimeric blastocysts were implanted into surrogate rats. Femur and tibia bones from P4-P10 chimeric pups were collected, and ESC-derived cells were isolated using FACS sorting for GFP. Subsequently, BM cells were injected into lethally irradiated recipient mice. **(B)** Donor ESC-derived cells were analyzed by FACS analysis of lung tissue on the fifth month after irradiation. Endothelial cells (Endo), hematopoietic cells (Hema), fibroblasts (Fib), epithelial cells (Epi), and pericytes (Peri) were identified in recipient lungs using specific cell surface markers. **(C)** Graph shows GFP fluorescence in lung cells 8 days (n = 10 mice per group) and 5 months (n = 14 mice per group) after BM transplantation. **(D)** Graph shows the proportions of endothelial cells, hematopoietic cells, fibroblasts, epithelial cells, and pericytes among total GFP + donor cells 5 months after BM transplantation. **(E)** Graph shows the proportions of GFP + cells in various populations of lung cells from recipient lungs 5 months after BM transplantation.

Mouse BM cells from mouse-rat chimeras provide long-term contribution to the lungs of mice injured by whole-body irradiation. Interspecies mouse-rat chimeras provide a unique environment which allows mouse-derived hematopoietic stem cells to accumulate in the bone marrow in large amounts (Wen et al., 2022). To determine whether mouse-rat BM contains progenitors capable of regenerating lung tissue, we examined lungs of syngeneic mice that underwent whole-body γ irradiation followed by BM transplantation. Mouse C57BL/6 ESCs labeled with green fluorescent protein (ESCs-GFP) were injected into rat SD blastocysts to generate mouse-rat chimeric embryos which were transferred to pregnant rats to undergo embryogenesis *in utero* (Figure 1A). After the birth of mouse-rat chimeras, BM was isolated from femurs and tibias. Mouse ESC-derived BM cells were FACS-sorted for GFP and injected into lethally irradiated syngeneic adult mice via the tail vein. Lung tissues were collected on the eighth day and the fifth month after BM cell transplantation (Figure 1A). To identify donor-derived cells in the lung, we used flow cytometry to detect GFP + cells in enzymatically digested lung tissue. Antibodies against mouse CD45, CD31, CD326, CD140a, and NG2 were used to identify hematopoietic cells (CD45^+^), endothelial cells (CD31^+^CD45^−^), epithelial cells (CD326+CD31^−^CD45^−^), fibroblasts (CD140a+CD31^−^CD45^−^CD326-) and pericytes (NG2+CD31^−^CD45^−^CD326-CD140a-) (Figure 1B, Supplementary Figure SE1, 2A). On the eighth day and the fifth month, percentages of GFP + cells among total lung cells were approximately 5% and 30% respectively (Figure 1C). Hematopoietic cells had the highest proportion among all GFP + donor cells, whereas the percentages of epithelial cells, endothelial cells, fibroblasts, and pericytes were lower (Figure 1D and E2C). The percentage of GFP + hematopoietic cells among total hematopoietic cells was the highest, reaching 40% at day 8 (Figure E2B-E2D) and 68% at 5 months (Figure 1E; Supplementary Figure SE2B). The contribution of donor BM cells to epithelial cells, endothelial cells, fibroblasts and pericytes was lower but easily detectable by FACS analysis of recipient lungs 5 months after BM transplantation (Figure 1E). Donor cells were not observed among non-hematopoietic cell types at day 8 (Supplementary Figure SE2D). Thus, donor BM cells from mouse-rat chimeras provide a long-term contribution to multiple respiratory cell types after radiation-induced lung injury.

**FIGURE 2:**
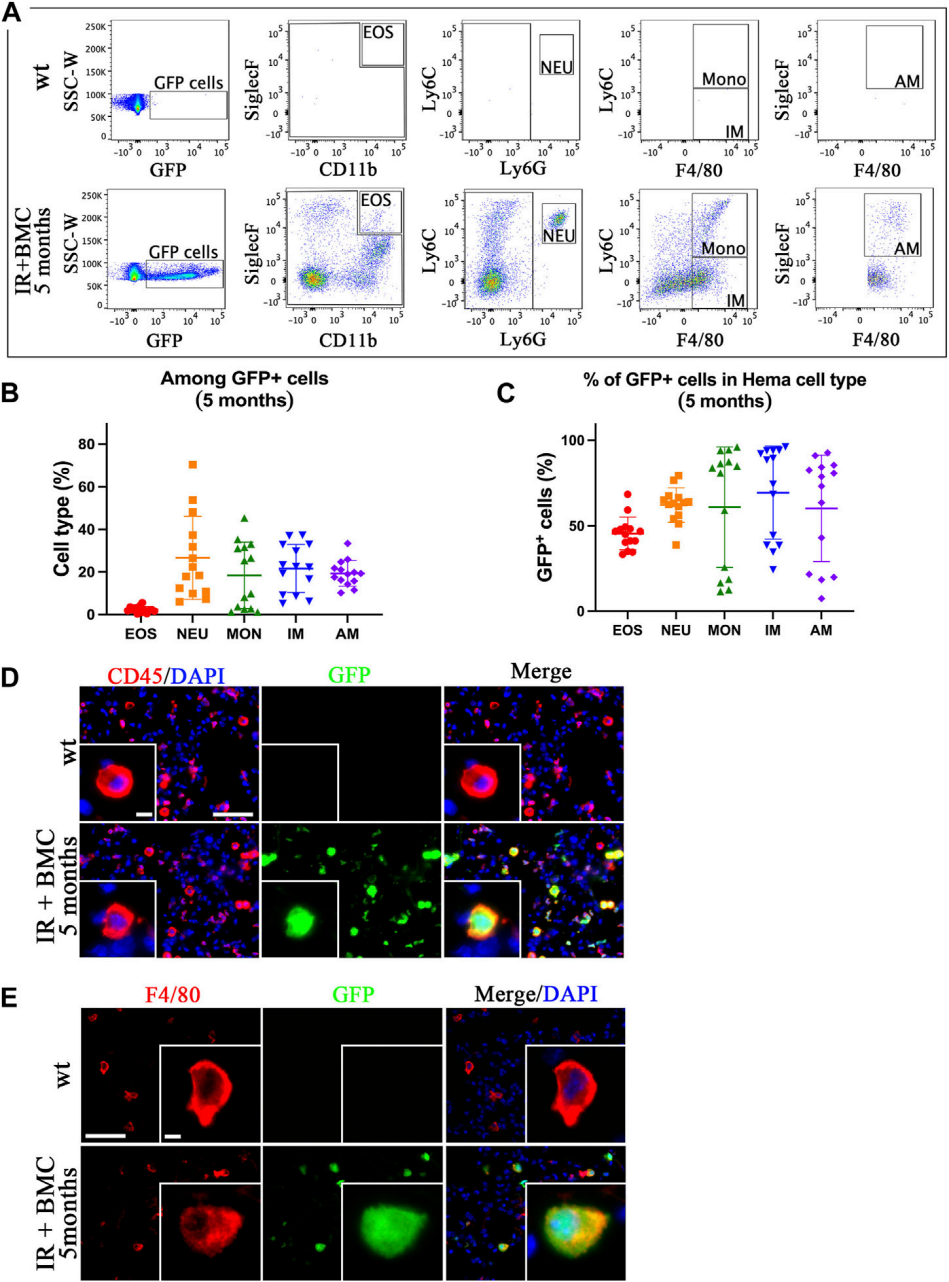
ESC-derived BM cells from mouse-rat chimeras contribute to various subtypes of hematopoietic cells in the lung tissue. **(A)** FACS analysis of hematopoietic cell subsets derived from donor BM cells in the recipient lungs 5 months after BM transplantation. Eosinophils (EOS), neutrophils (NEU), monocytes (Mono), interstitial macrophages (IM), and alveolar macrophages (AM) were identified in the lungs of recipient mice using flow cytometry. **(B)** Graph displays the proportions of eosinophils, neutrophils, monocytes, interstitial macrophages, and alveolar macrophages among total GFP + donor cells in recipient mice lungs 5 months after BM transplantation. **(C)** Graph shows the proportions of GFP + cells in various hematopoietic cell types isolated from the lung tissue 5 months after BM transplantation. **(D, E)** Donor hematopoietic BM cells contribute to CD45^+^ and F4/80+ cells in recipient lungs 5 months after BM transplantation. The lung sections were stained for CD45 and F4/80 (red). GFP was used to identify donor-derived cells (green). Cell nuclei were counterstained with DAPI (blue). Scale bars in D are 50 μm and 5 µm (insert). Scale bars in E are 50 μm and 4 µm (insert).

Mouse BM cells from mouse-rat chimeras reconstitute lung hematopoietic cells after whole-body irradiation. To identify donor BMC-derived hematopoietic cell types in the lungs of irradiated mice, the lungs were harvested on the eighth day and the fifth month after BM cell transplantation, enzymatically digested, and used for FACS to identify eosinophils (EOS) (CD45^+^CD11b+SiglecF+), neutrophils (NEU) (CD45+Ly6G + Ly6C+), monocytes (MONO) (CD45+Ly6G-Ly6C + F4/80+), interstitial macrophages (IM) (CD45+Ly6G-Ly6C-F4/80+), and alveolar macrophages (AM) (CD45+SiglecF + Ly6G-Ly6C-F4/80+) (Supplementary Figure SE3, 4). GFP was detected in all hematopoietic cell types in the lungs of irradiated mice that received BMC transplantation (Figures 2A–C; Supplementary Figure SE4B-D). The proportion of BMC-derived eosinophils among GFP + cells was lower compared to other cell types (Figure 2B; Supplementary Figure SE4C). The long-term contribution of donor BM cells to all hematopoietic cell populations in the lung tissue was approximately 50% as demonstrated by FACS analysis (Figure 2C). In contrast, the short-term contribution after 8 days of donor BM cells was higher in neutrophils compared to other cell types (Supplementary Figure SE4D). Immunostaining of frozen lung tissues for CD45, F4/80, and CD3e showed co-localization of GFP with markers of hematopoietic cells (CD45), macrophages (F4/80) and T cells (CD3e) (Figures 2D,E; Supplementary Figure SE5). GFP was not detected in uninjured mice without BM cell transplantation (Figure 2D, E; Supplementary Figure SE5). These results indicate that donor BM cells from mouse-rat chimeras effectively contribute to hematopoietic cell types in the lung tissue after radiation-induced injury.

**FIGURE 3:**
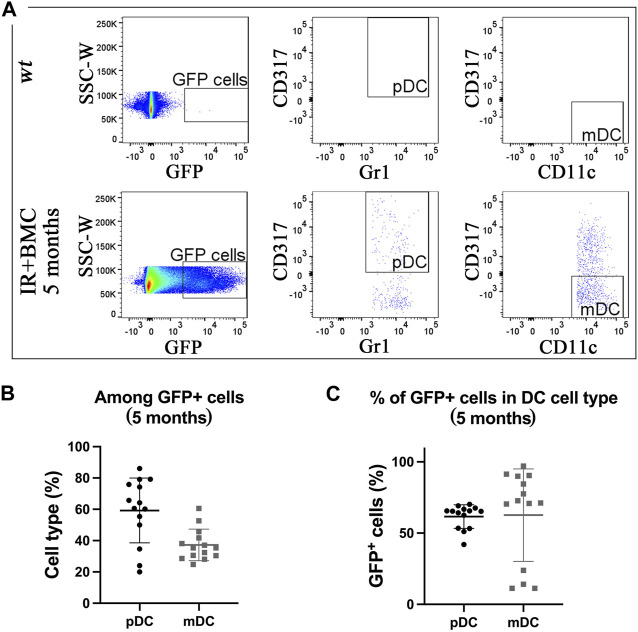
Donor BM cells from mouse-rat chimeras contribute to two subtypes of pulmonary dendritic cells. **(A)** FACS analysis of dendritic cell (DC) subsets derived from mouse GFP-ESCs in recipient lungs 5 months after BM transplantation. Myeloid dendritic cells (mDC) and plasmacytoid dendritic cells (pDC) were identified using multiple cell surface markers. **(B)** Graph displays the proportions of pDC and mDC among total GFP + donor DCs obtained from recipient lungs 5 months after BM transplantation. **(C)** The graph shows the proportions of GFP + cells in pDC and mDC cell subsets in recipient lungs 5 months after BM transplantation.

Mouse BM cells from mouse-rat chimeras provide long-term contribution to lung resident dendritic cells after RILI. To determine if donor BM-derived cells contribute to lung resident dendritic cells (DCs), flow cytometry was used to identify myeloid dendritic cells (mDCs) (CD45^+^CD11b+CD317-SiglecF-CD11c+Gr1-) and plasmacytoid dendritic cells (pDCs) (CD45^+^CD11b-CD317+CD11c+Gr1+) in the lung tissue (Figure E6A-B). GFP + donor cells were detected in both pDCs and mDCs in lungs of mice that received BM transplantation (Figures 3A–C). The proportion of donor-derived pDCs among total GFP + dendritic cells was higher compared to mDCs for both long-term and short-term BMC contributions (Figure 3B; Supplementary Figure SE6D-E). Donor-derived cells accounted for more than 50% of pDCs and mDCs by 5 months after BM cell transplantation (Figure 3C; Supplementary Figure SE6E). Thus, donor BM cells from mouse-rat chimeras effectively contribute to dendritic cell types after radiation-induced injury.

**FIGURE 4:**
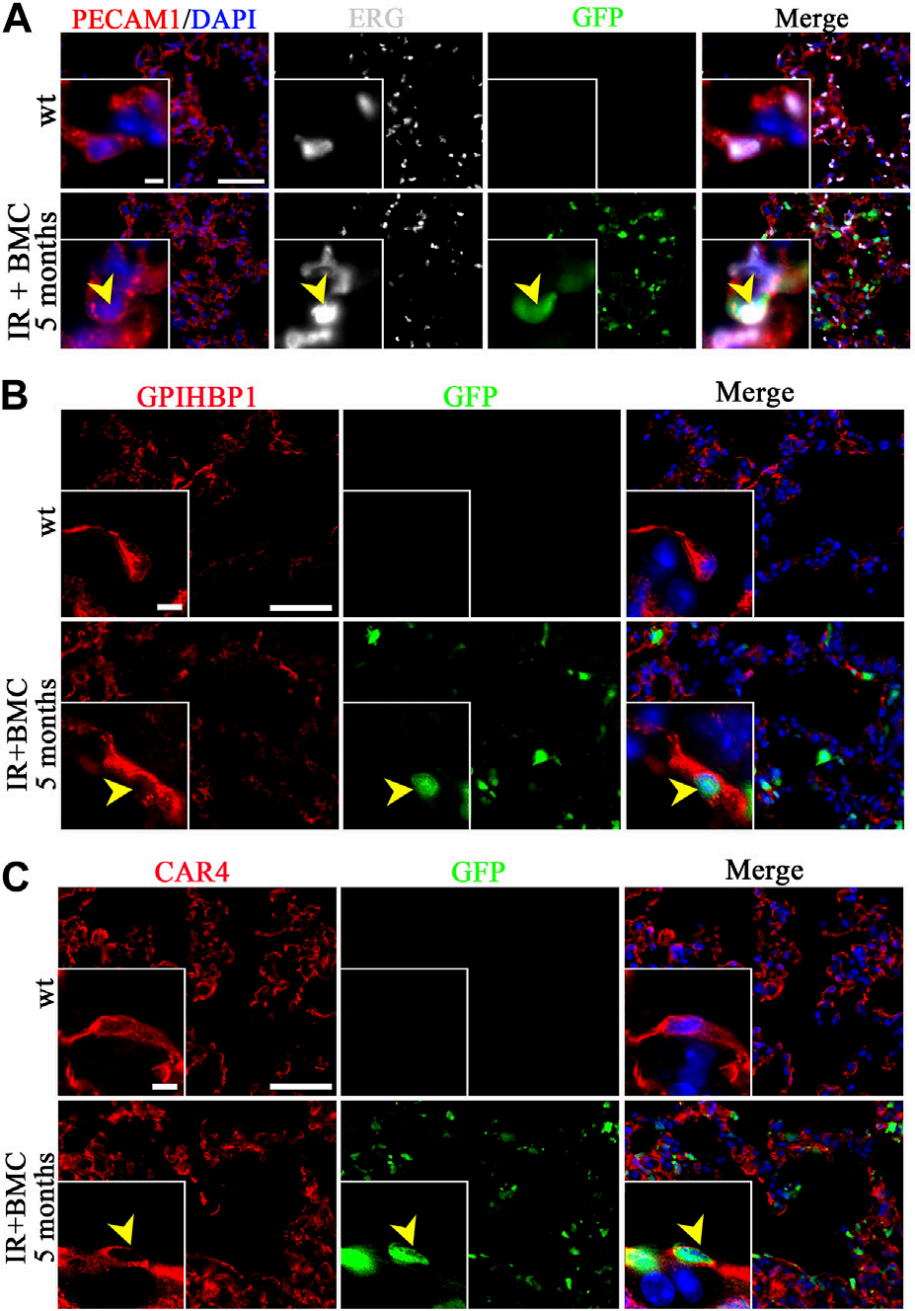
Donor BM cells from mouse-rat chimeras contribute to lung endothelial cells of mice 5 months after BM transplantation. **(A)** Immunostaining of frozen lung sections for PECAM1 (red) and ERG (white) reveals extensive microvascular networks in the lungs of recipient mice 5 months after BM transplantation. Donor ESC-derived cells are detected using GFP (green). Sections were counterstained with DAPI (blue). Scale bars: 50 μm and 5 μm (inserts). **(B, C)** Donor BM-derived cells (BMC) contribute to gCAPs and aCAPs in the lungs of recipient mice 5 months after BM transplantation. gCAPs were identified using immunostaining for GPIHBP1, whereas aCAPs were stained for CAR4. Scale bars: 50 μm and 5 μm (insert).

Donor BM cells from mouse-rat chimeras contribute to lung stromal, endothelial and epithelial cells after RILI. We used immunostaining to identify the location of BM-derived cells in the recipient lung because flow cytometry showed a relatively small but consistent contribution of BM-derived donor cells to pulmonary endothelial, epithelial, and stromal cell lineages (Figure 1E). GFP-labeled endothelial cells co-expressing PECAM1 (CD31) and endothelial-specific transcription factor ERG were detected in alveolar regions of recipient mice 5 months after BM cell transplantation (Figure 4A). Donor-derived endothelial cells also expressed VE-cadherin (Supplementary Figure SE7). Donor-derived endothelial cells were detected in veins but not arteries of recipient mice 5 months after BM transplantation (Supplementary Figure SE8, 9). GFP-labeled cells were observed in both general capillary cells (gCAP or CAP1) and alveolar capillary cells (aCAP or CAP2) as demonstrated by immunostaining for gCAP marker GPIHBP1 and aCAP marker CAR4 (Figures 4B,C). Lung pericytes marked by PDGFRb (Supplementary Figure SE10) and NG2 (Supplementary Figure SE10B) also contained donor-derived GFP + cells. Interestingly, ATI cells expressing T1α, and ATII cells expressing Pro-SPC were found among GFP + cells in lungs of mice 5 months after BM transplantation (Supplementary Figure SE11A). In the airway epithelium, GFP + club cells were rare, as demonstrated by co-localization of GFP with club cell marker CCSP (Supplementary Figure SE11B). Donor BM cells contributed to lung fibroblasts expressing Vimentin (Supplementary Figure SE12A) and PDGFRa (Supplementary Figure SE12B). Altogether, both flow cytometry and immunostaining demonstrated that donor mouse BM cells from mouse-rat chimeras have a capacity to differentiate into multiple respiratory cell types in the lungs of mice injured by whole-body irradiation. In summary, BM cells contribute to various stromal and vascular cells in the lungs of irradiated mice that underwent BM transplantation.

**FIGURE 5:**
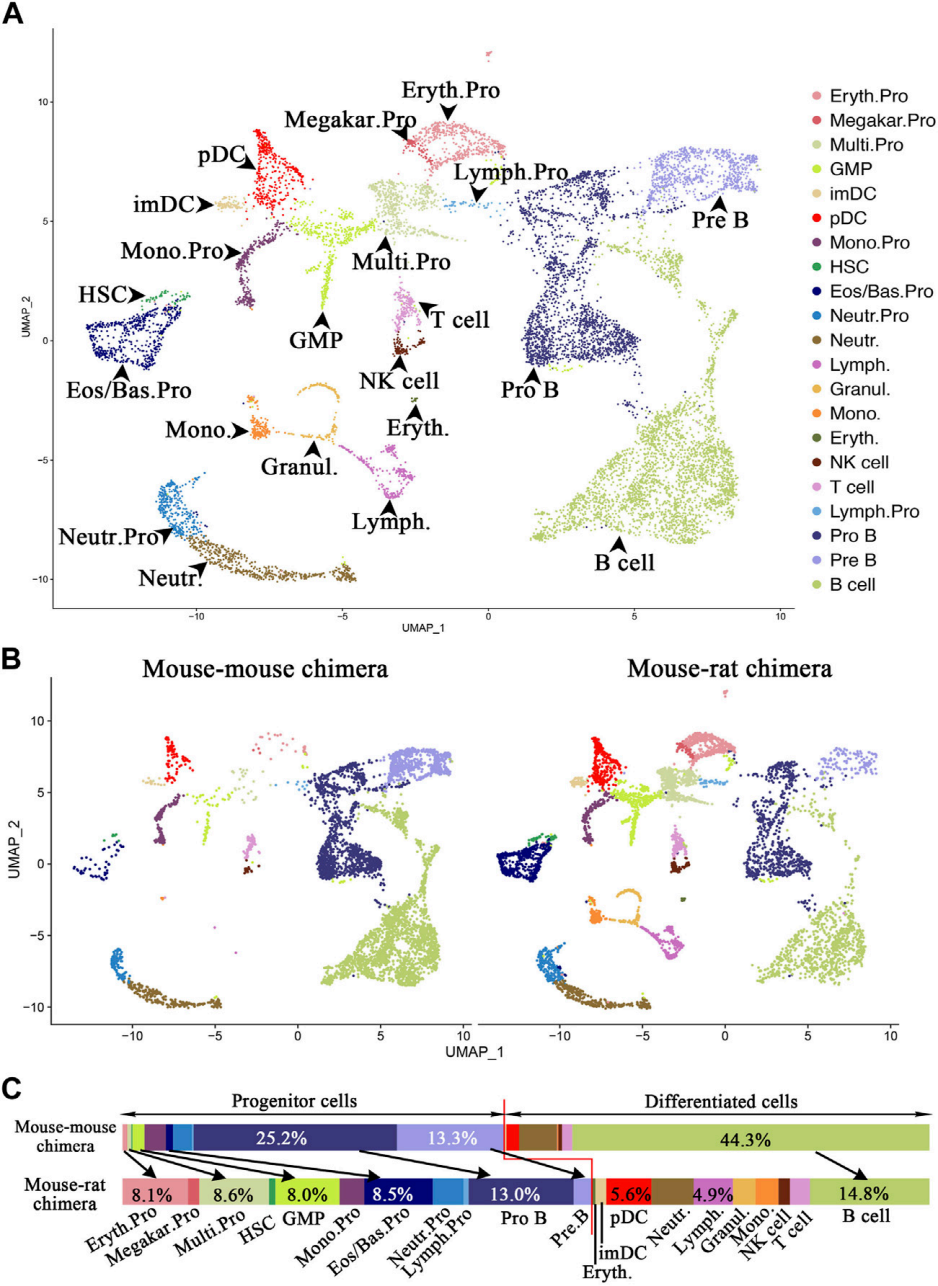
Single-cell RNA sequencing identifies ESC-derived BM cell subtypes in mouse-rat chimeras. **(A)** Uniform manifold approximation and projection (UMAP) plot shows identical hematopoietic cell clusters in BM cells from mouse-rat and mouse-mouse (control) chimeras. ESC-derived BM cells were obtained from P10 chimeras using FACS sorting for GFP + cells. Cells from n = 3 animals per group were pooled prior to FACS sorting. Cells are colored according to cell subtype. **(B)** UMAP plots of single cells from chimeras. The left panel shows GFP + cells from control mouse-mouse chimeras (n = 5,282 cells), while the right panel shows GFP + cells from mouse-rat chimeras (n = 5,945 cells). Cells are colored based on cell subtypes. **(C)** Comparison of cell proportions in individual cell clusters between mouse-mouse and mouse-rat chimeras. The percentages of progenitor cells are higher in mouse-rat chimeras compared to mouse-mouse chimeras. Cell types and abbreviations: Erythroid progenitor (Eryth.Pro), Megakaryocyte progenitor (Megakar.Pro), Multipotent progenitor (Multi.Pro), Granulocyte-monocyte progenitor (GMP), Immature DC (ImDC), Plasmacytoid DC (pDC), Monocyte progenitor (Mono.Pro), Hematopoietic stem cell (HSC), Eosinophil/Basophil progenitor (Eos/Bas.Pro), Neutrophil progenitor (Neutr.Pro), Neutrophil (Neutr.), Lymphoid cell (Lymph.), Granulocyte (Granul.), Monocyte (Mono.), Erythrocyte (Eryth.), NK cell, T cell, Lymphoid progenitor (Lymph.Pro), Pro B cell, Pre B cell, and B cell.

Single-cell RNA sequencing identifies multiple ESC-derived cell subsets in the bone marrow of mouse-rat chimeras. To identify ESC-derived progenitor cells in the BM of mouse-rat chimeras that can differentiate into respiratory cell types, we FACS-sorted GFP + BM cells and performed scRNAseq using 10X Chromium platform. Mouse ESC-derived cells from mouse-rat chimeras at postnatal day 10 (P10) were compared with ESC-derived cells from mouse-mouse chimeras at the same age (control group). Control mouse-mouse chimeras were generated by injection of mouse blastocysts with the same line of mouse ESC-GFP cells to control for embryonic manipulations, genetic background and pluripotency of ESC line used for blastocyst injection. Since the number of HSCs and other progenitor cells in the bone marrow is relatively low, we mixed 90% of FACS-sorted GFP + Lin-progenitor cells with 10% of GFP + Lin + differentiated cells to enrich for BM progenitor cells before scRNAseq. Since BM was washed from the bone, the vast majority of cells were hematopoietic. To account for individual variations, BM cells from three chimeras in each group were pooled prior to FACS sorting. Based on published gene expression signatures in mouse BM cells (Wen et al., 2022), 11,227 cells from 21 major cell subtypes were identified, including erythroid progenitors, megakaryocytes, multipotent progenitors, monocytes, granulocyte-monocyte progenitors, eosinophil/basophil progenitors, neutrophil progenitor cells, lymphoid progenitor cells, pro-B cells, pre-B cells, immature DCs, plasmacytoid DCs, hematopoietic stem cells, lymphoid cells, granulocytes, monocytes, erythrocytes, NK cells, T cells, and B cells (Figure 5A; 5B, and E13). Compared to the control chimeras, the percentage of progenitor cells was higher in BM of mouse-rat chimeras (Figure 5B). Specifically, the percentages of mouse ESC-derived erythroid progenitors (8.1%), multipotent progenitors (8.6%), granulocyte-monocyte progenitors (8.0%), eosinophil/basophil progenitors (8.5%), and hematopoietic stem cells (0.8%) were higher in mouse-rat chimeras compared to control mouse-mouse chimeras (Figures 5B, C). Among progenitor cells, only pro-B and pre-B cells were lower in mouse-rat chimeras compared to control mouse-mouse chimeras (Figure 5C). In contrast, B cells were more abundant in BM of control chimeras compared to mouse-rat chimeras (Figures 5B, C). The percentages of DCs, monocytes, NK cells, and T cells were higher in the mouse-rat chimeras (Figure 5C). Overall, although ESC-derived BM cells in both the mouse-rat and control chimeras exhibited similar cell compositions, the mouse-rat chimeras were enriched with various progenitor cells.

Gene expression characteristics of ESC-derived progenitor cells, such as multipotent progenitor cells, monocyte progenitor cells, eosinophils/basophils progenitor cells, pro-B cells, and pre-B cells, were highly similar between mouse-rat and control mouse-mouse chimeras as shown by the heatmaps (Supplementary Figure SE14). Multipotent progenitor cells in both groups expressed *Ctla2a*, *Cd34* and *Adgrl4,* whereas monocyte progenitor cells expressed *Clec4a3*, *Cybb* and *S100a4* (Supplementary Figure SE14). These findings are consistent with the known markers of these cells (Nestorowa et al., 2016). Eosinophils/basophils progenitor cells expressed *Mcpt8*, *Prss34*, and *Cpa3*; neutrophil progenitors were positive for *Cstdc4*, *Stfa211*, and *Asprv1*; pro-B cells were marked by *Vpreb3*, *Cd79a*, and *Ebf1*; pre-B cells expressed *Igkc*, *My14*, and *H1f5* in both experimental groups (Supplementary Figure SE14). Thus, although gene expression signatures of ESC-derived progenitor cells were highly similar between mouse-rat and mouse-mouse chimeras, the BM of the mouse-rat chimeras contained more ESC-derived progenitor cells.

**FIGURE 6:**
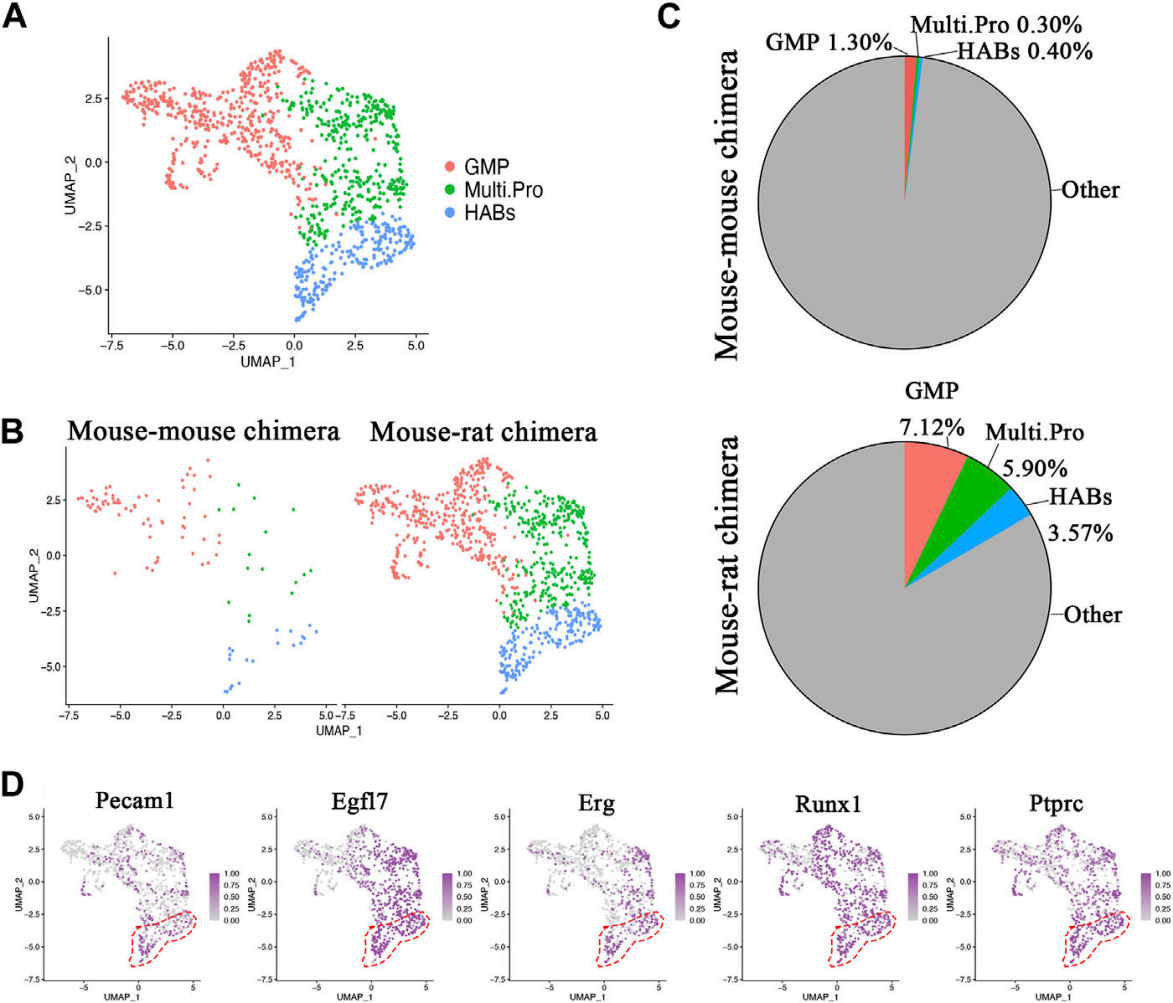
Single-cell RNA sequencing identifies ESC-derived endothelial progenitor cells in BM of mouse-rat chimeras. **(A)** UMAP dimensional reduction plot from scRNAseq of P10 BM cells shows the clustering of granulocyte-monocyte progenitor cells, multipotent progenitor cells and hemangioblasts-like cells (HABs). BM cells from n = 3 mice in each group were pooled together prior to the scRNAseq analysis. **(B)** Split dimensional reduction plots of granulocyte-monocyte progenitor cells, multipotent progenitor clusters and HABs from mouse-mouse chimeras (n = 106 cells) and mouse-rat chimeras (n = 986 cells). **(C)** Comparison of the proportions of granulocyte-monocyte progenitor cells, multipotent progenitor cells and HABs between mouse-mouse and mouse-rat chimeras. The percentages of HABs, granulocyte-monocyte progenitor cells and multipotent progenitor cells are higher in mouse-rat chimeras compared to mouse-mouse chimeras. **(D)** Scatter plots show the expression of *Pecam1*, *Egfl7*, *Erg*, *Runx1*, and *Ptprc* mRNA in cell clusters. Dashed line (red) indicates HABs. Cell types and abbreviations: Multipotent progenitor cells (Multi.Pro), Granulocyte-monocyte progenitor cells (GMP), Hemangioblasts-like cells (HABs).

Single cell RNA sequencing identifies ESC-derived endothelial progenitor cells in mouse-rat BM. Immunostaining of lung sections (Figures 4A–C) and flow cytometry of lung cell suspensions (Figure 1B; Supplementary Figure SE1D-E) showed that BM cells from mouse-rat chimeras contribute to lung endothelial cells of irradiated mice. To determine whether endothelial progenitor cells are present in donor BM cells from mouse-rat chimeras, sub-clustering analysis was performed using several closely related progenitor populations, including granulocyte-monocyte progenitor cells, multipotent progenitor cells, and the cells co-expressing endothelial mRNAs (Pecam1, Erg) and hematopoietic mRNAs (Ptprc (CD45), Runx1) that we termed, “BM hemangioblasts” or HABs (Figures 6A–D). HABs exhibited remarkable similarities of gene expression signature with embryonic hemangioblasts (Pijuan-Sala et al., 2019; Mittnenzweig et al., 2021). BM and HABs shared similar marker genes with hemangioblasts isolated from mouse E7.0 embryos such as *F2r*, *Id3* and *Runx1* (Supplementary Table SE1). However, the difference in *Fli1*, *Kdr* and *Pecam1*, clearly distinguished BM HABs from embryonic hemangioblasts (Supplementary Table SE1).

While mouse-rat chimeras contained 986 cells (432 granulocyte-monocyte progenitors, 351 multipotent progenitors and 212 BM HABs), the control mouse-mouse chimeras contained only 106 cells (69 granulocyte-monocyte progenitors, 16 multipotent progenitors and 21 BM HABs) (Figure 6B). The proportion of these progenitor cells among ESC-derived BM cells was higher in mouse-rat chimeras compared to mouse-mouse controls (Figure 6C; Supplementary Figure SE15). *Runx1*, a marker of early hematopoietic progenitor cells, that derive from hemogenic endothelium in dorsal aorta (Chen et al., 2009), was present in all 3 cell types. In addition to Runx1, BM HABs expressed endothelial mRNAs, including *Pecam1*, *Egfl7*, and *Erg* (Figure 6D). The heatmap showed remarkable similarities in gene expression signatures of granulocyte-monocyte progenitors, multipotent progenitors and HABs derived from mouse ESCs in mouse-rat and control mouse-mouse chimeras (Supplementary Figure SE16). Multipotent progenitor cells in both experimental groups expressed *Hlf* and *Adgr14*, whereas granulocyte-monocyte progenitor cells expressed *Ctsg*, *Prtn3*, Mpo, *Elane* and *S100a8,* consistent with normal expression patterns in these BM cells. In addition to endothelial and hematopoietic genes, BM HABs expressed *Hes1*, *Dntt*, *Ebf1, Crip1* and *Smad7* (Supplementary Figure SE16). Altogether, mouse ESCs have a higher propensity to differentiate into multiple cell progenitors including HABs during BM development. Gene expression signatures of these ESC-derived cells are highly similar regardless of the host species in which ESCs underwent differentiation.

**FIGURE 7:**
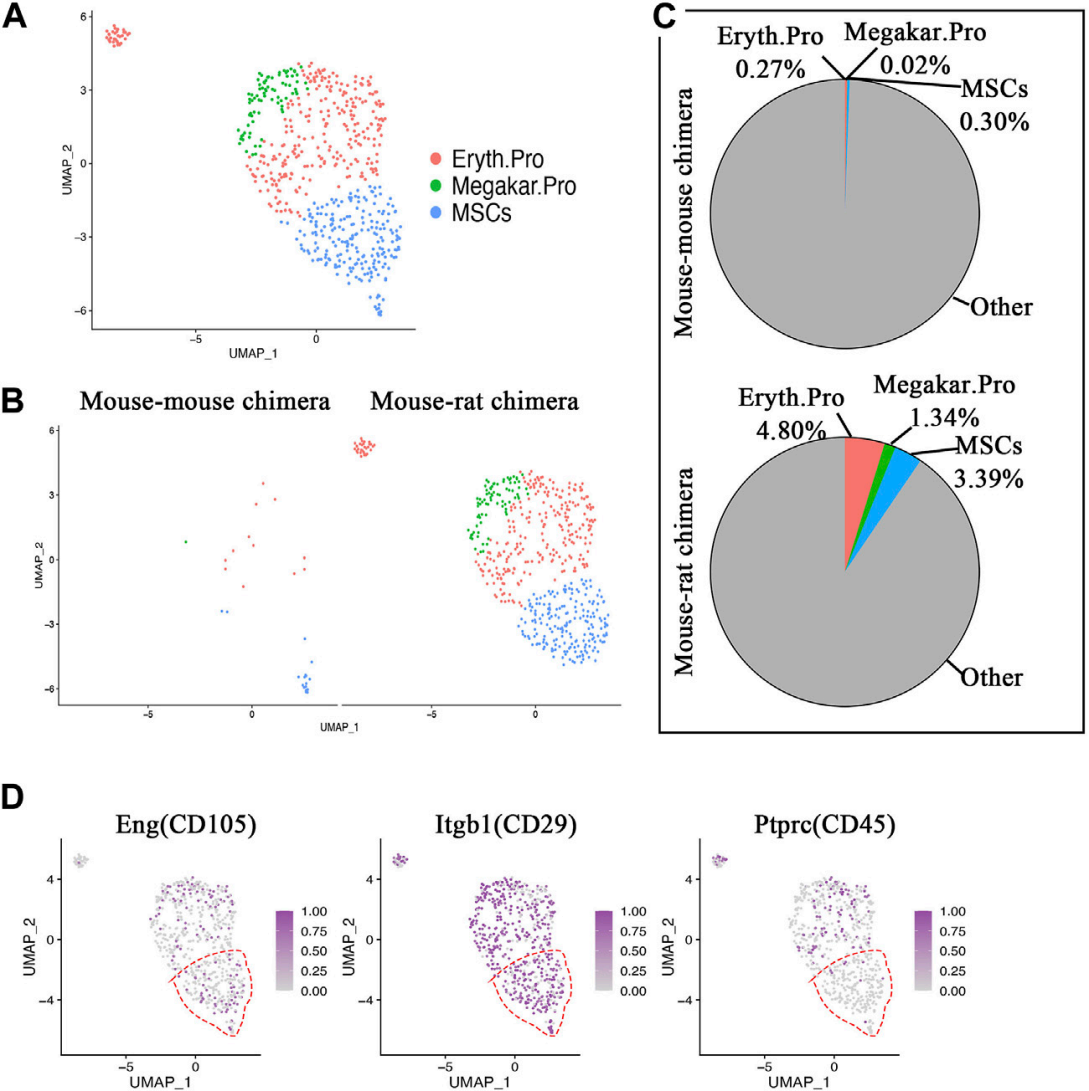
Single-cell RNA sequencing identifies ESC-derived mesenchymal stromal cells in BM of mouse-rat chimeras. **(A)** UMAP dimensional reduction plot from scRNAseq of P10 BM cells shows the clustering of erythroid progenitor cells, megakaryocyte progenitor cells and mesenchymal stromal cells (MSCs). BM cells from n = 3 mice in each group were pooled together prior to the scRNAseq analysis. **(B)** Split dimensional reduction plots of erythroid progenitor cells, megakaryocyte progenitor cells and MSCs from mouse-mouse chimeras (n = 31 cells) and mouse-rat chimeras (n = 566 cells). **(C)** Comparison of the proportions of erythroid progenitor cells, megakaryocyte progenitor cells and MSCs between mouse-mouse and mouse-rat chimeras. The percentages of MSCs, erythroid progenitor cells and megakaryocyte progenitor cells are higher in mouse-rat chimeras compared to mouse-mouse chimeras. **(D)** Scatter plots show the expression of *Eng, Itgb1* and *Ptprc*. In MSC cluster (red dashed line), *Eng* and *Itgb1* are expressed, while *Ptprc* is absent. Cell types and abbreviations: Erythroid progenitor cells (Eryth.Pro), Megakaryocyte progenitor cells (Megakar.Pro), Mesenchymal stromal cells (MSCs).

Single-cell RNA sequencing identifies ESC-derived mesenchymal stromal cells in the bone marrow of the mouse-rat chimeras. Immunostaining of lung sections and flow cytometry showed that BM cells from mouse-rat chimeras contribute to epithelial cells, fibroblasts, and pericytes in irradiated recipient lungs (Figure 1B; Supplementary Figure SE1D-E), raising a possibility that donor BM cells contain progenitors of these cell lineages. Therefore, we followed up by performing a sub-clustering analysis of scRNAseq to identify ESC-derived BM mesenchymal stromal cells (MSCs) that are known to differentiate into multiple mesenchymal cell lineages, including fibroblasts and pericytes (Wolock et al., 2019; Leimkuhler et al., 2021). BM MSCs exhibited gene expression similarities with erythroid progenitor cells and megakaryocyte progenitor cells as shown by UMAP plot (Figure 7A). Interestingly, erythrocyte progenitors, megakaryocyte progenitors and MSCs accounted for only 31 cells in control mouse-mouse chimeras, including 14 erythroid progenitor cells, one megakaryocyte progenitor cell and 16 MSCs (Figure 7B). In contrast, mouse-rat chimeras contained 565 cells from these cell types, including 285 erythroid progenitor cells, 80 megakaryocyte progenitor cells and 201 MSCs (Figures 7B, C). ESC-derived MSCs from mouse-rat chimeras exhibited a gene expression signature similar to previously identified MSCs signature (GSE132151) (Supplementary Table SE12).

Using scRNAseq UMAP analysis, BM MSCs were examined for the presence of typical BM MSC markers CD29, and CD105, and the absence of hematopoietic markers CD45 (Figure 7D), consistent with published studies (Zhang et al., 2018). Heatmaps showed high similarity in gene expression signatures between erythroid progenitors, megakaryocyte progenitors and MSCs in mouse-rat and mouse-mouse chimeras (Supplementary Figure SE17). Consistent with published studies (Wolock et al., 2019), erythroid progenitor cells expressed *Plac8* and *Vim*, megakaryocyte progenitor cells expressed *Pf4* and *Plek,* and BM MSCs expressed *Cpox*, *Blvrb*, *Ermap*, *Alad*, *Hspd1*, *Glrx5* and *Nolc1* (Supplementary Figure SE17). Interestingly, we did not detect epithelial progenitor cells among ESC-derived BM cells that were used for BM transplantation in irradiated mice, as demonstrated by the absence of cells expressing markers of pulmonary epithelial progenitors such as *Nkx2-1* and *Sftpc* (SPC) (Supplementary Figure SE18). *Sftpb* (SPB), and *Ager* mRNA were also undetectable (Supplementary Figure SE18). In summary, the bone marrow of mouse-rat chimeras provides a reservoir of mouse ESC-derived progenitor cells with regenerative capacity toward multiple respiratory cell lineages injured by γ-radiation.

## Discussion

In this manuscript, we examined the lung regenerative potential of bone marrow from mouse-rat chimeras, the artificial animals generated by injection of rat blastocysts with mouse pluripotent embryonic stem cells (Wen et al., 2022). Published studies demonstrate that BM from mouse-rat chimeras is capable of regenerating all hematopoietic cell lineages in the blood and bone marrow of lethally irradiated mice. While a multi-lineage engraftation by a single BM hematopoietic stem cell from normal BM has been demonstrated for the respiratory system (Krause et al., 2001), HSC engraftment is inefficient and mostly occurs through a fusion of hematopoietic progenitor cells with lung-resident epithelial, endothelial and stromal cells, but not through direct differentiation of HSCs into respiratory cell lineages (Wagers and Weissman, 2004). An important contribution of this study is that the bone marrow of mouse-rat chimeras contains progenitors of lung endothelial cells, fibroblasts and pericytes that are rare in normal mouse bone marrow. Although donor BM cells from mouse-rat chimeras contribute to lung epithelial cells including AT1, AT2 and club cell, based on immunostaining and flow cytometry of lung tissue, we were unable to find respiratory epithelial progenitors expressing *Nkx2-1* and *Sftpc* in the bone marrow of mouse-rat chimeras by scRNAseq. It is possible that the epithelial progenitors are ultra-rare and were not captured by our scRNAseq. Another possibility is that lung epithelial progenitors arise after interaction of donor BM cells with the lung tissue environment. Finally, GFP + epithelial cells can be a product of cell fusion between donor BM cells and lung-resident epithelial cells, consistent with published studies showing the fusion of BM cells with various resident cell types (Wagers and Weissman, 2004).

Our results showed that the proportion of mouse ESC-derived hematopoietic progenitor cells in mouse-rat BM is considerably higher compared to control mouse-mouse chimeras. This could be due to the early development of mouse HSCs in mouse-rat embryos compared to rat HSCs as was found in recent studies (Wen et al., 2022). Mouse embryos develop faster than rat embryos by approximately 1.5 days (Wen et al., 2022), and mouse HSAs in mouse-rat chimeras arise faster from the dorsal aorta, leading to faster colonization of the fetal liver and BM by mouse HSCs compared to rat HSCs (Wen et al., 2022). Mouse HSCs from BM cells of mouse-rat chimeras are functional because they can rescue syngeneic mice exposed to lethal doses of γ-radiation (Wen et al., 2022). Since mouse BM cells from mouse-rat chimeras regenerate all hematopoietic cell, lineages in the blood and BM (Wen et al., 2022), it is not surprising that donor BM cells can contribute to all hematopoietic cells that reside in the lung tissue given that whole-body irradiation is a catastrophic injury which causes extensive lung damage. Our findings that a majority of lung-resident alveolar macrophages and dendritic cells derive from donor BM cells suggest that the whole-body irradiation depletes a pool of lung-resident hematopoietic progenitors leading to differentiation of donor BM cells into alveolar macrophages and dendritic cells that are normally self-maintained in the quiescent adult lung (Hashimoto et al., 2013). Our data is consistent with published studies showing that BM cells can differentiate into alveolar macrophages after lung injury (Bernard et al., 2012).

Published studies have shown that intravenous delivery of BM-derived endothelial progenitor cells attenuates lipopolysaccharide-induced lung injury and improves survival in rats (Mao et al., 2010). Consistent with these studies, donor BM cells from mouse-rat chimeras contributed to the long-term regeneration of several endothelial cell types in the lung tissue, including gCAPs, aCAPs and venous cells. Since gCAP cells contain progenitors for all lung endothelial cell types (Gillich et al., 2020), the expansion and differentiation of donor BM-derived gCAPs can explain significant numbers of donor-derived endothelial cells in recipient mice injured by γ-radiation. Our scRNAseq analysis of donor BM cells suggests that endothelial progenitor cells in mouse-rat bone marrow can be bipotential because they share a gene expression signature with yolk sac hemangioblasts which are known to differentiate into endothelial and hematopoietic cell lineages (Huber et al., 2004). Our findings are consistent with previous studies showing that normal BM cells can contribute to lung endothelial cells in irradiated mice after BM transplantation (Raoul et al., 2007). Although it is unclear why donor BM cells contribute to lung fibroblasts and pericytes, it is possible that mesenchymal stromal cells within donor BM cells could be their progenitors. BM MS differentiated into multiple mesenchymal lung lineages after lung injury (Yamada et al., 2004). Our data demonstrate that ESC-derived BM cells generated in mouse-rat chimeras are capable of differentiating into many respiratory cell types within the lung-specific microenvironment after lethal γ-radiation.

Although BM of mouse-rat chimeras develops from a mixture of mouse and rat cells, mouse BM cells from mouse-rat chimeras cannot be distinguished from normal mouse BM cells based on cellular composition of the bone marrow and gene expression signatures of BM cell types. One limitation of our studies is that the differentiation capacity of mouse-rat BM cells was established from whole BM transplants rather than transplantation of purified MSCs or BM hemangioblasts. Since the numbers of these cells are limited, these experiments are technically challenging and require the development of efficient purification methods to isolate these cells from mouse-rat chimeras. Future studies are needed to examine the transplantation of purified BM hemangioblasts and MSCs into irradiated mice and to investigate whether chimeric cells regenerate endothelial and mesenchymal cell types and improve outcomes after radiation injury.

In summary, the bone marrow of mouse-rat chimeras is enriched with mouse hematopoietic progenitor cells, mesenchymal stromal cells and hemangioblasts-like cells that can differentiate into multiple respiratory cell lineages after whole-body irradiation. The bone marrow of interspecies chimeras represents an “*in vivo* reservoir” of ESC-derived lung progenitor cells. Our studies can be useful for future differentiation of patient-derived iPSCs in the bone marrow of large animals to generate patient-specific MSCs and hemangioblasts for lung regenerative medicine.

## Data Availability

Previously published Single cell RNA sequencing data from BMC of P10 Mouse-mouse chimera and Mouse-rat chimera that were reanalyzed here are available under accession code GSE184940. Single cell mRNA sequencing of stromal cell from mouse bone marrow (GSE132151) and the mouse Yolk Sac dataset generated by Pijuan-Sala et al. (https://github.com/MarioniLab/EmbryoTimecourse2018) were used in this study.
