# Supplementary material for: The bone marrow of mouse-rat chimeras contains progenitors of multiple pulmonary cell lineages
Source: Front Cell Dev Biol. 2024 Apr 17;12:1394098. doi: 10.3389/fcell.2024.1394098 (PMC11061410; doi:10.3389/fcell.2024.1394098)

Supplementary Material Presentation

The Bone Marrow of Mouse-Rat chimeras contains progenitors of multiple pulmonary cell lineages

Enhong Li[1], Bingqiang Wen[1], Dengfeng Gao[2], Timothy R. Kalin[3], Guolun Wang[4], Tanya V. Kalin[1,5], and Vladimir V. Kalinichenko[1,6*]

[1] Phoenix Children's Research Institute, Department of Child Health, University of Arizona College of Medicine-Phoenix, Phoenix, AZ, 85004, USA

[2] State Key Laboratory of Animal Biotech Breeding, College of Biological Sciences, China Agricultural University, Beijing, 100091, China

[3] College of Arts and Sciences, University of Cincinnati, Cincinnati, OH, 45221, USA

[4] Division of Pulmonary Biology, Cincinnati Children's Hospital Medical Center, Cincinnati, OH, 45229, USA

[5] Center for Cancer and Blood Diseases, Phoenix Children's Hospital, Phoenix, AZ, 85016, USA

[6] Division of Neonatology, Phoenix Children's Hospital, Phoenix, AZ, 85016, USA

***Correspondence to**: Vladimir V. Kalinichenko, Email: vkalin@arizona.edu

Phoenix Children's Research Institute, University of Arizona College of Medicine-Phoenix, 475 N. 5th Street, Biomedical Sciences Partnership Building (BSPB), Phoenix, AZ, 85004. Tel.: 602-827-2078.

Table E1 **:** The gene expression of HABs in BM cells compare with the HABs of E7.0 Yolk Sac. The Wilcoxon Rank Sum test implemented via the function *FindAllMarkers* in Seurat package was used for data analysis. Similar gene expression is shown by bold font, n.d. is not detected.

Gene			BMC-HABs		YS-HABs	
SYMBOL	GENE NAME	GenBank	FC	P-value	FC	P-value
Ccnd2	**cyclin D2**	NM_009829.3	1.31242332	0.066	2.78612035	3.90E-176
Inka1	**inka box actin regulator 1**	NM_026597.4	1.20745403	0.018	2.58795298	0
Tal1	**T cell acute lymphocytic leukemia 1**	NM_011527.3	1.11928282	1	2.54463375	0
Ifitm1	**interferon induced transmembrane protein 1**	NM_001360727.1	1.67398145	0.048	2.06407217	1.61E-123
Egfl7	**EGF-like domain 7**	NM_001164564.2	2.02812742	2.14E-28	1.83032414	1.31E-53
S100a10	**S100 calcium binding protein A10 (calpactin)**	NM_009112.2	1.29627476	0.211	1.82202714	1.13E-74
F2r	**coagulation factor II (thrombin) receptor**	NM_010169.4	1.16289979	0.000	1.78656612	6.37E-142
Runx1	**runt related transcription factor 1**	NM_009821.3	1.11928282	1	1.19705423	7.21E-13
Tpm4	**tropomyosin 4**	NM_001001491.2	1.47581734	3.32E-10	1.76722977	4.69E-98
Hmga2	**high mobility group AT-hook 2**	NM_001347170.1	1.52995443	1.15E-12	1.75973338	1.37E-71
Id3	**inhibitor of DNA binding 3**	NM_008321.2	1.51729325	1.92E-16	1.6164351	1.08E-50
Junb	**jun B proto-oncogene**	NM_008416.3	1.79527097	4.65E-08	1.59162841	3.05E-67
Dusp2	**dual specificity phosphatase 2**	NM_010090.2	2.20568482	4.38E-22	1.50136144	0
Ppp1r15a	**protein phosphatase 1, regulatory subunit 15A**	NM_008654.2	1.65640441	3.11E-11	1.57095594	2.86E-70
Cnn3	**calponin 3, acidic**	NM_028044.2	1.41763454	3.64E-06	1.30428686	9.95E-23
Fli1	Friend leukemia integration 1	NM_001410252.1	n.d.	n.d.	1.42418365	0.00E+00
Kdr	kinase insert domain protein receptor	NM_001403143.1	n.d.	n.d.	1.86742196	0.00E+00
Pecam1	platelet/endothelial cell adhesion molecule 1	NM_001032378.2	1.2158151	0.001	n.d.	n.d.
Ptprc	protein tyrosine phosphatase, receptor type, C	NM_001268286.2	1.4049337	3.20E-07	n.d.	n.d.
Erg	ETS transcription factor	NM_001302183.1	1.42342352	2.21E-10	n.d.	n.d.

Table E2 **:** The gene expression of MSC in BM cells compare with the MSC in GSE132151 BM cells. The Wilcoxon Rank Sum test implemented via the function *FindAllMarkers* in Seurat package was used for data analysis. Similar gene expression is shown by bold font, n.d. is not detected.

Gene			BMC-MSC		GSE132151-MSC	
SYMBOL	Gene Name	GenBank	FC	P-Value	FC	P-Value
Slc29a1	**solute carrier family 29 (nucleoside transporters), member 1**	NM_001376987.1	1.5353261	2.50E-14	3.00502065	4.37E-88
Aplp2	**amyloid beta (A4) precursor-like protein 2**	NM_001102456.2	1.4507390	5.55E-10	2.23395037	9.53E-47
Mt2	**metallothionein 2**	NM_008630.2	4.5811308	1.12E-51	1.90002489	4.97E-21
Kpnb1	**karyopherin (importin) beta 1**	NM_008379.3	1.3771719	7.68E-08	1.83866366	8.99E-15
Imp3	**IMP3, U3 small nucleolar ribonucleoprotein**	NM_133976.2	1.3838140	3.61E-11	1.81799285	7.86E-06
Tubb5	**tubulin, beta 5 class I**	NM_011655.5	1.3816183	3.49E-12	1.80016815	2.50E-20
Col5a1	**collagen, type V, alpha 1**	NM_015734.2	1.4018854	2.94E-06	1.58258462	4.23E-10
Tmem14c	**transmembrane protein 14C**	NM_001360770.1	1.6372367	5.41E-20	1.52944779	7.25E-06
Eif4g2	**eukaryotic translation initiation factor 4, gamma 2**	NM_001040131.2	1.3618504	1.61E-11	1.5954787	3.03E-11
Canx	**calnexin**	NM_001110500.1	1.3690952	3.63E-12	1.67312593	1.99E-14
Mat2a	**methionine adenosyltransferase II, alpha**	NM_001363799.1	1.5239838	3.07E-13	1.65843953	2.43E-10
Hspa8	**heat shock protein 8**	NM_031165.5	1.4369689	5.21E-22	2.3969431	2.60E-75
Ybx1	**Y box protein 1**	NM_011732.2	1.1986667	9.78E-19	1.86519848	2.50E-20
Slc25a4	**solute carrier family 25 (mitochondrial carrier, adenine nucleotide translocator), member 4**	NM_007450.5	1.2862253	8.25E-06	1.64331684	6.42E-18
Necap2	**NECAP endocytosis associated 2**	NM_025383.4	1.5046136	8.98E-17	1.44738829	0.000
Nop10	**NOP10 ribonucleoprotein**	NM_025403.4	1.4231285	1.39E-20	1.4615614	9.63E-05
Lpl	lipoprotein lipase	NM_008509.2	n.d.	n.d.	4.46960653	1.07E-176
Kitl	kit ligand	NM_013598.3	n.d.	n.d.	4.4463826	3.20E-148
Cxcl12	chemokine (C-X-C motif) ligand 12	NM_001012477.2	n.d.	n.d.	4.1676538	4.35E-205
Car1	carbonic anhydrase 1	NM_009799.4	2.1015210	3.78E-10	n.d.	n.d.
Tfrc	transferrin receptor	NM_001357298.1	1.9124587	3.09E-21	n.d.	n.d.

Table E3 : Antibodies used for Flow Cytometry (FC) and Immunofluorescence staining (IF).

Antibody	Method	Manufacturer	Catalog No.	Dilution
CD11b	FC	BD Biosciences	552850	1:100
CD11c	FC	Biolegend	117339	1:100
CD31	FC	Thermo Fisher	48-0311-82	1:100
CD45	FC	eBioscience	56-0451-82	1:100
CD45	FC	eBioscience	47-0451-82	1:100
CD68	FC	Biolegend	137009	1:100
CD140a	FC	eBioscience	25-1401-82	1:100
CD317	FC	Biolegend	127015	1:100
CD326	FC	eBioscience	17-5791-82	1:100
F4/80	FC	eBioscience	48-4801-82	1:100
Fc Block	FC	Biolegend	101320	1:100
Gr1	FC	Biolegend	108433	1:100
Ly6C	FC	Biolegend	128037	1:100
Ly6G	FC	Biolegend	127641	1:100
NG2	FC/IF	Millipore	AB5320C3	1:100
SiglecF	FC	Biolegend	155505	1:100
7-AAD	FC	Biolegend	420404	1:100
Car4	IF	R&D	AF2414	1:200
CCSP	IF	Seven Hills Bioreagents	WRAB-3950	1:500
CD3e	IF	Santa Cruz	sc-1127	1:200
CD45	IF	R&D	AF114	1:200
F4/80	IF	Biolegend	122602	1:200
Gpihbp1	IF	Thermo Fisher	PA5-16976	1:200
Pecam1	IF	BD Bioscience	553370	1:200
Pdgfra	IF	R&D	AF1062	1:300
Pdgfrb	IF	Cell signaling	3169	1:200
Pro-SPC	IF	Seven Hills Bioreagents	WRAB-9337	1:500
T1a	IF	University of lowa Hybridoma bank	DSHB 8.1.1.	1:500
Vimentin	IF	Santa Cruz	sc-7557	1:300

Figure E1. Identification of endothelial cells, hematopoietic cells, fibroblasts, epithelial cells, and pericytes in irradiated mouse lungs. FACS gating strategy shows the identification of GFP+, endothelial cells (Endo), hematopoietic cells (Hema), fibroblasts (Fib), epithelial cells (Epi), and pericytes (Peri) in irradiated mouse lungs 5 months after BM transplantation.

Figure E2. Flow cytometry identifies various cell types in irradiated mouse lungs 8 days and 5 months after BM transplantation. (A) Lung samples were collected from untreated mice (control), lethally irradiated mice without BM transplantation (IR), and lethally irradiated mice with BM transplantation (IR+BMC). BM transplantation was performed using ESC-derived BM cells obtained from juvenile mouse-rat chimeras. FACS analysis was conducted 8 days and 5 months after BM transplantation. **(B)** Histograms show the presence of donor-ESC-derived (GFP+) endothelial cells (Endo), hematopoietic cells (Hema), fibroblasts (Fib), epithelial cells (Epi), and pericytes (Peri) in recipient lungs after BM transplantation (green line). Lung samples of mice without BM transplantation were used to identify autofluorescence in the GFP channel (blue line). Lung samples were collected 8 days and 5 months after BM transplantation and subjected to FACS analysis. **(C)** Graph shows the proportions of endothelial cells, hematopoietic cells, fibroblasts, epithelial cells, and pericytes in the total GFP+ cells 8 days after BM transplantation (n=10). **(D)** Graph shows the proportions of GFP+ cells in various cell populations 8 days after BM transplantation (n=10).

Figure E3. Identification of eosinophils, neutrophils, monocytes, interstitial macrophages, and alveolar macrophages in recipient mouse lungs after BM transplantation. FACS gating strategy shows the identification of GFP+, eosinophils (EOS), neutrophils (NEU), monocytes (MONO), interstitial macrophages (IM), and alveolar macrophages (AM) in recipient mouse lungs 5 months after BM transplantation.

Figure E4. FACS identifies eosinophils, neutrophils, monocytes, interstitial macrophages, and alveolar macrophages in lungs of lethally irradiated mice 8 days and 5 months after BM transplantation. (A) Lung samples were collected from untreated mice (control), lethally irradiated mice without BM transplantation (IR), and lethally irradiated mice with BM transplantation (IR+BMC). BM transplantation was performed using ESC-derived BM cells obtained from juvenile mouse-rat chimeras. FACS analysis was conducted 8 days and 5 months after BM transplantation. **(B)** Identification of donor ESC -derived hematopoietic cells in recipient lungs after BM transplantation. Histograms show the presence of GFP+ eosinophils (EOS), neutrophils (NEU), monocytes (Mono), interstitial macrophages (IM), and alveolar macrophages (AM) in recipient mouse lungs after BM transplantation (green line). Lung samples from mice without BM transplantation were used to identify autofluorescence in the GFP channel (blue line). Lung samples were collected 8 days and 5 months after BM transplantation for FACS analysis. **(C)** The graph displays the proportions of eosinophils, neutrophils, monocytes, interstitial macrophages, and alveolar macrophages in the total GFP+ cell population 8 days after BM transplantation. **(D)** Graph shows the proportions of GFP+ cells in various cell subsets in recipient lungs 8 days after BM transplantation.

Figure E5. Immunostaining for CD3e shows that donor BM cells contribute to T cells. CD3e-positive T cells (red) are observed in lung sections of recipient mice that underwent BM transplantation. Donor ESC-derived cells are detected with GFP (green). Nuclei are counterstained with DAPI (blue). The scale bars are 50 μm and 5 μm (insert).

Figure E6. Donor ESC-derived cells contribute to dendritic cells in lungs of lethally irradiated mice after BM transplantation. (A) Identification of myeloid dendritic cells (mDC) and plasmacytoid dendritic cells (pDC). FACS gating strategy shows the identification of mDC and

pDC in lungs of wild-type mice. **(B)** FACS analysis identifies mDC and pDC after BM transplantation. Lung samples were collected from untreated mice (wt), lethally irradiated mice without BM transplantation (IR), and lethally irradiated mice with BM transplantation (IR+BMC). BM transplantation was performed using ESC-derived BM cells obtained from juvenile mouse-rat chimeras. FACS analysis was performed 8 days and 5 months after BM transplantation. **(C)** Identification of donor ESC-derived cells in lungs of irradiated mice after BM transplantation. Histograms show the presence of donor-derived (GFP+) mDC and pDC in the lung of irradiated mice after BM transplantation (green line). Lung samples were collected 8 days and 5 months after BM transplantation for FACS analysis. **(D)** Graph displays the proportions of pDC and mDC in the total population of GFP+ cells 8 days after BM transplantation. (E) Graph shows the proportions of GFP+ cells in pDC and mDC populations 8 days after BM transplantation.

Figure E7. Donor BM cells contribute to mature alveolar endothelial cells 5 months after BM transplantation. Immunostaining of frozen lung sections for VE-cadherin (red) reveals mature endothelial cells in pulmonary alveoli of recipient mice 5 months after BM transplantation. Donor ESC-derived cells are detected using GFP (green). Sections were counterstained with DAPI (blue). Scale bars: 50 μm and 5 μm (inserts).

Figure E8. Donor BM cells do not contribute to endothelial cells of large artery 5 months after BM transplantation. Immunostaining of frozen lung sections for PECAM1 shows the lake of donor cells among endothelial cells in pulmonary arteries of irradiated mice 5 months after BM transplantation. GFP was used to identify donor-derived cells. Cell nuclei were counterstained with DAPI (blue). Cell types and abbreviations: Art, arteries; Br, bronchioles. Scale bars are 100 μm and 50 μm.

Figure E9. Donor BM cells contribute to pulmonary veins of irradiated mice 5 months after BM transplantation. Immunostaining of frozen lung sections for PECAM1 shows the presence of donor cells among venous endothelial and stromal cells in lungs of irradiated mice 5 months after BM transplantation. GFP was used to identify donor ESC-derived cells. Cell nuclei were counterstained with DAPI (blue). Cell type and abbreviations: Br, bronchioles. Scale bars are 100 μm and 50 μm.

Figure E10. Donor BM cells contribute to pericytes of irradiated lungs 5 months after BM transplantation. (A-B) Immunostaining of frozen lung sections for PDGFRb and NG2 shows the presence of pericytes in lungs of irradiated mice 5 months after BM transplantation. GFP was used to identify donor ESC-derived cells. Cell nuclei were counterstained with DAPI (blue). Scale bars are 50 μm and 5 μm (insert).

Figure E11. Donor BM cells contribute to lung epithelial cells 5 months after BM transplantation. (A) Immunostaining of frozen lung sections shows the expression of T1α and Pro-SPC in alveolar epithelial cells. Pro-SPC-positive ATII cells (white) and T1α-positive ATI cells (red) are present in peripheral lung regions. Donor ESC-derived cells are detected with GFP (green). High magnification images of ATII cells (white arrow heads) and ATI cells (yellow arrow heads) are shown in inserts. Sections were counterstained with DAPI (blue). Scale bars are 50 μm and 5 μm (inserts). **(B)** GFP+ club cells in the bronchiolar epithelium. Club cells were stained with CCSP. Slides were counterstained with DAPI. Donor-derived cells were detected with GFP (green). Scale bars are 100 μm (low magnification images), 50 μm (high magnification images), and 5 μm (inserts).

Figure E12. Donor BM cells contribute to pulmonary fibroblasts in irradiated mice 5 months after BM transplantation. Immunostaining of frozen lung sections for VIMENTIN (A) and

PDGFRa (B) shows the presence of fibroblasts in the lungs of irradiated mice 5 months after BM transplantation. GFP was used to identify donor-derived cells. Nuclei were counterstained with DAPI (blue). Scale bars are 100 μm (low magnification images), 50 μm (high magnification images), and 5 μm (inserts).

Figure E13. Single-cell RNA sequencing identifies hematopoietic cell subsets in BM of mouse-rat chimeras. The integrated projection of ESC-derived BM cells from mouse-rat and mouse-mouse (control) chimeras. Cells were obtained from the bone marrow of P10 chimeras. Cell clusters were identified using the Uniform Manifold Approximation and Projection (UMAP) method. Expression of marker genes reveals distinct hematopoietic BM cell clusters.

Figure E14. ESC-derived progenitor cells in mouse-rat and mouse-mouse chimeras exhibit similar gene expression profiles. The heatmap demonstrates significant similarities in gene expression signatures of multipotent progenitor cells (Multi. Pro), monocyte progenitor cells (Mono. Pro), eosinophils/basophils progenitor cells (Eos/Bas. Pro), neutrophil progenitor cells (Neutr. Pro), Pro B cells, and Pre-B cells obtained from mouse-rat (MR) and mouse-mouse (MM) chimeras. scRNAseq was performed using BM cell suspensions that were FACS-sorted for GFP. The left column shows marker genes for various cell subsets.

Figure E15. Single-cell RNAseq analysis identifies ESC-derived endothelial progenitor cells in mouse-mouse and mouse-rat chimeras. Scatter plots show the expression of *Pecam1*, *Egfl7*, *Kit*, *Fli1*, *Erg*, *Tal1*, *Cd34*, *Runx1*, and *Ptprc* in mouse-mouse and mouse-rat chimeras.

Figure E16. ESC-derived granulocyte-monocyte progenitor cells, multipotent progenitor cells and BM hemangioblasts exhibit similar gene expression profiles in mouse-rat and mouse-mouse chimeras. The heatmap reveals significant similarities in gene expression signatures of granulocyte-monocyte progenitor (GMP), multipotent progenitor (Multi. Pro) and hemangioblasts (HABs) obtained from mouse-rat (MR) and mouse-mouse (MM) chimeras. scRNAseq was performed using BM cell-suspensions that were FACS-sorted for GFP.

Figure E17. ESC-derived erythroid progenitor cells, megakaryocytes progenitor cells and BM mesenchymal stromal cells exhibit similar gene expression profiles in mouse-rat and mouse-mouse chimeras. The heatmap demonstrates significant similarities in gene expression signatures of erythroid progenitor (Eryth. Pro), megakaryocytes progenitor (Megakar. Pro) and mesenchymal stromal cells (MSCs) obtained from mouse-rat (MR) and mouse-mouse (MM) chimeras. scRNAseq was performed using BM cell suspensions that were FACS-sorted for GFP.

Figure E18. Single-cell RNAseq analysis shows the lack of cells expressing lung epithelial gene in GFP+ BM cell population. Scatter plots show the absence of *Nkx2-1*, *Sftpc*, *Sftpb* and *Ager* mRNA between mouse-mouse and mouse-rat chimeras.

Figure E1

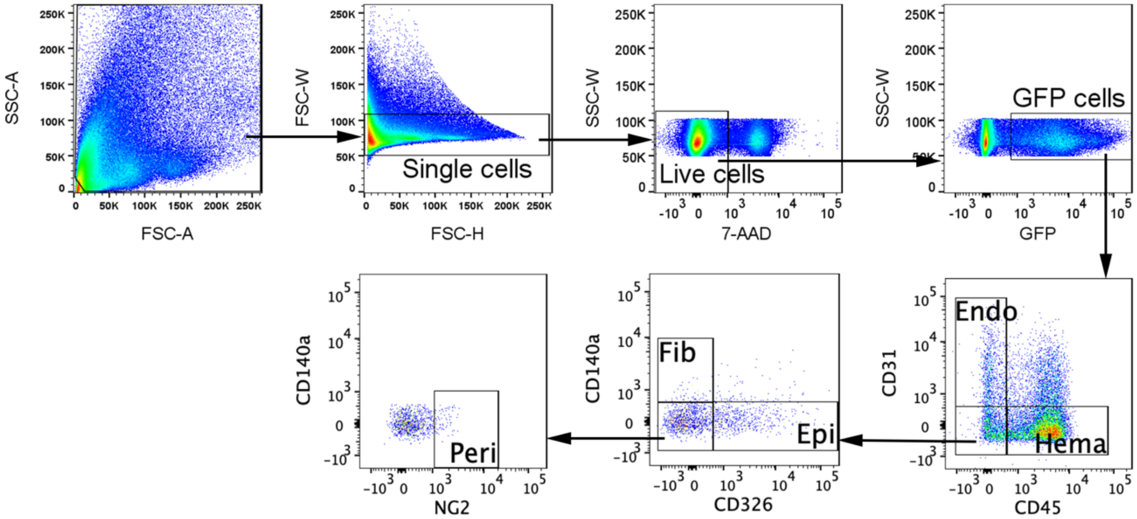
SSC-A
FSC-A
FSC-W
FSC-H
Single cells
SSC-W
7-AAD
Live cells
SSC-W
GFP
GFP cells
CD31
CD45
Endo
Hema
CD140a
CD326
Fib
Epi
CD140a
NG2
Peri

Figure E2

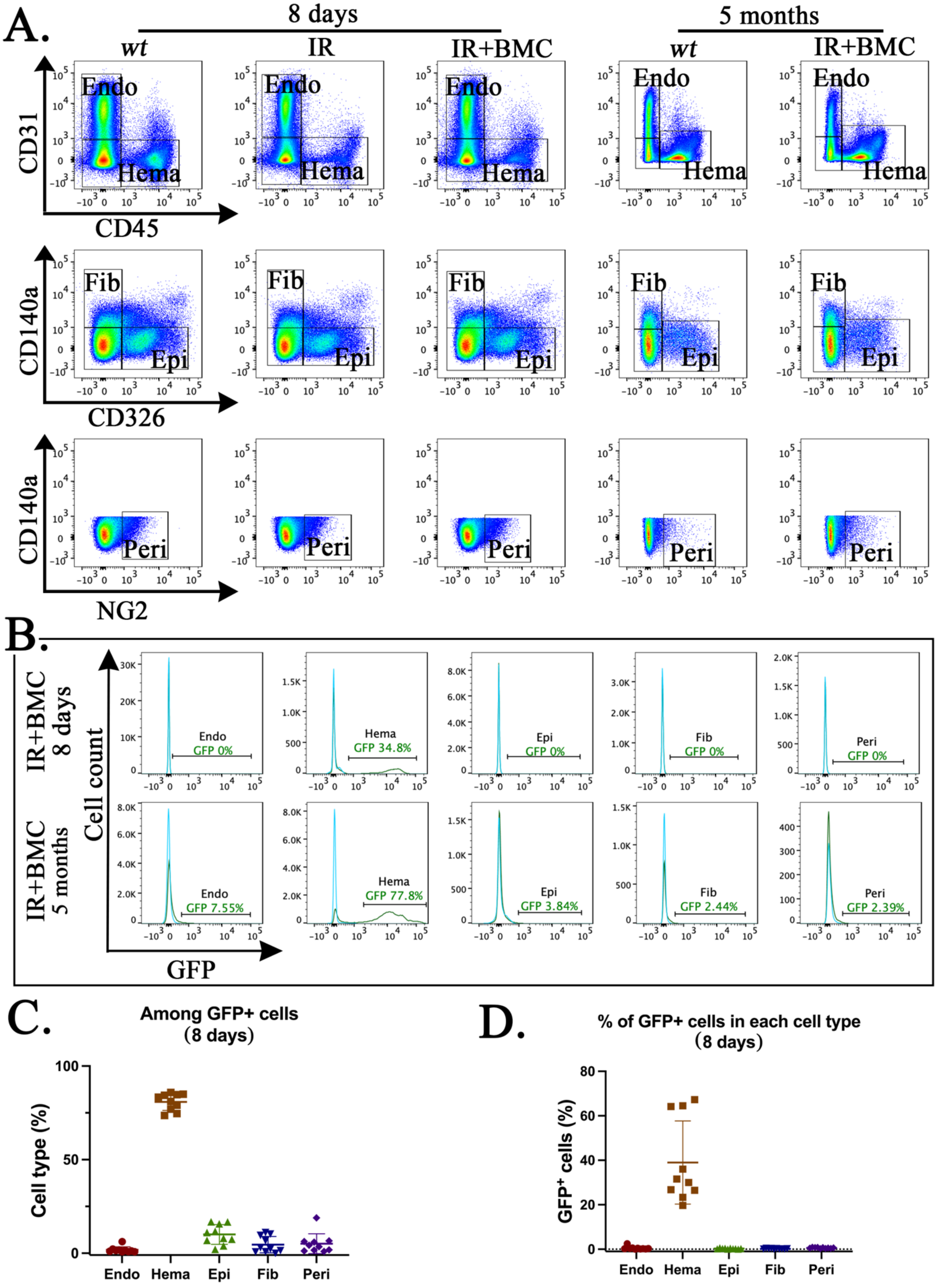
A.
8 days
5 months
wt
IR
IR+BMC
wt
IR+BMC
CD31
CD45
Endo
Hema
CD140a
CD326
Fib
Epi
CD140a
NG2
Peri
B.
IR+BMC 8 days
IR+BMC 5 months
Cell count
GFP
Endo GFP 0%
Hema GFP 34.8%
Epi GFP 0%
Fib GFP 0%
Peri GFP 0%
Endo GFP 7.55%
Hema GFP 77.8%
Epi GFP 3.84%
Fib GFP 2.44%
Peri GFP 2.39%
C.
Among GFP+ cells (8 days)
Cell type (%)
Endo
Hema
Epi
Fib
Peri
D.
% of GFP+ cells in each cell type (8 days)
GFP+ cells (%)
Endo
Hema
Epi
Fib
Peri

Figure E3

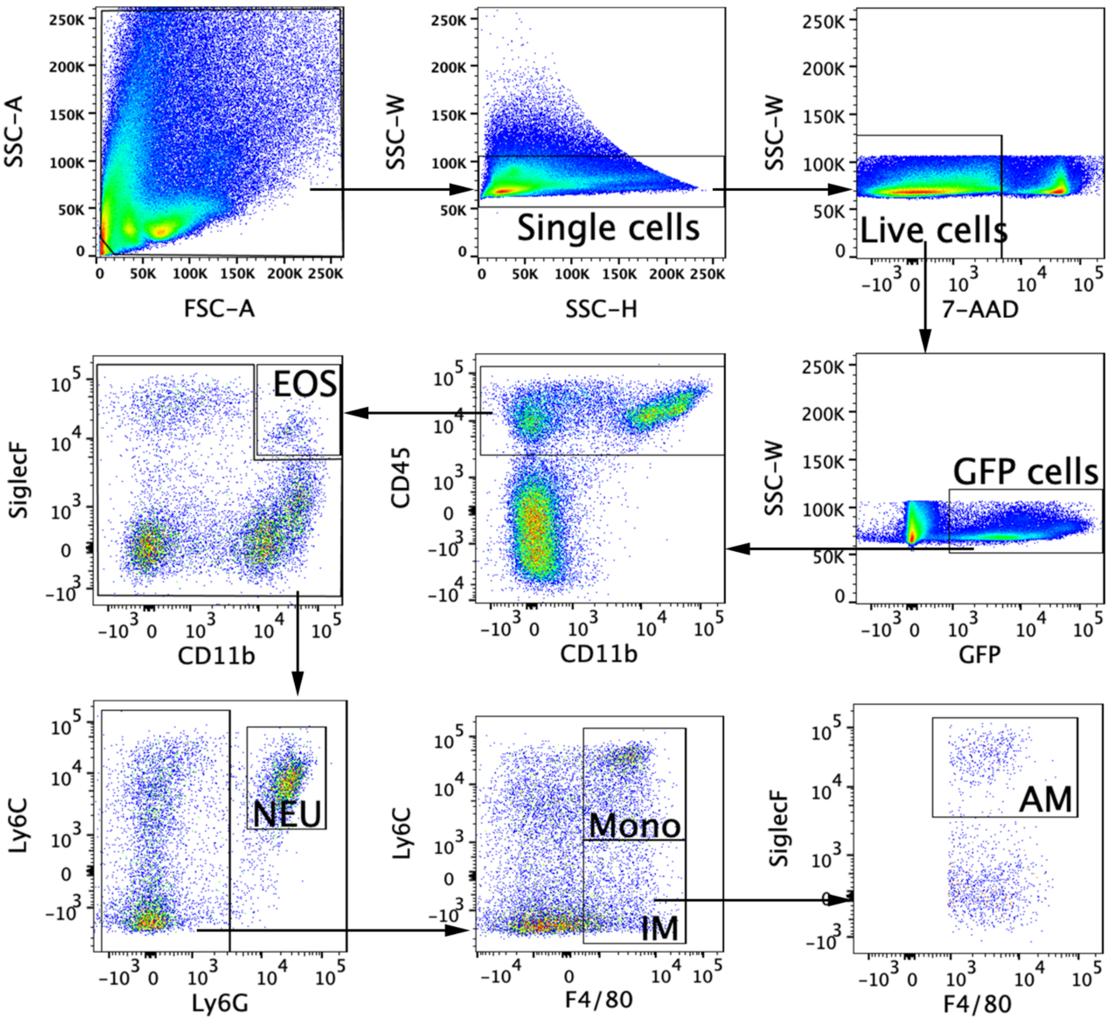
SSC-A
FSC-A
SSC-W
SSC-H
Single cells
SSC-W
7-AAD
Live cells
SSC-W
GFP
GFP cells
CD45
CD11b
SiglecF
CD11b
EOS
Ly6C
Ly6G
NEU
Ly6C
F4/80
Mono
IM
SiglecF
F4/80
AM

Figure E4

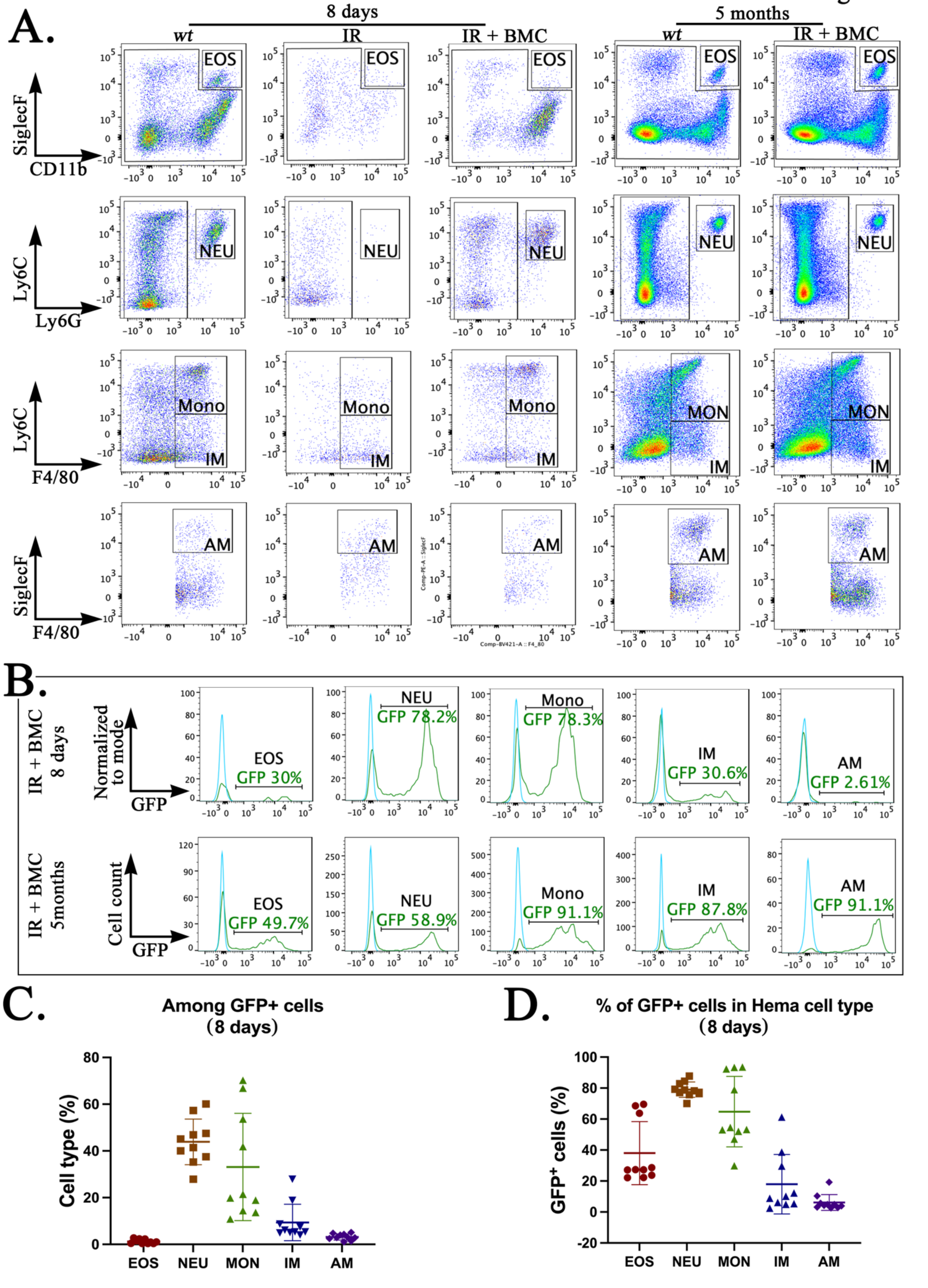
A.
8 days
5 months
wt
IR
IR + BMC
SiglecF
CD11b
EOS
Ly6C
Ly6G
NEU
F4/80
Mono
IM
MON
AM
B.
IR + BMC 8 days
IR + BMC 5months
Normalized to mode
Cell count
GFP
EOS
GFP 30%
NEU
GFP 78.2%
Mono
GFP 78.3%
IM
GFP 30.6%
AM
GFP 2.61%
EOS
GFP 49.7%
NEU
GFP 58.9%
Mono
GFP 91.1%
IM
GFP 87.8%
AM
GFP 91.1%
C.
Among GFP+ cells
(8 days)
Cell type (%)
EOS
NEU
MON
IM
AM
D.
% of GFP+ cells in Hema cell type
(8 days)
GFP⁺ cells (%)
EOS
NEU
MON
IM
AM

Figure E5

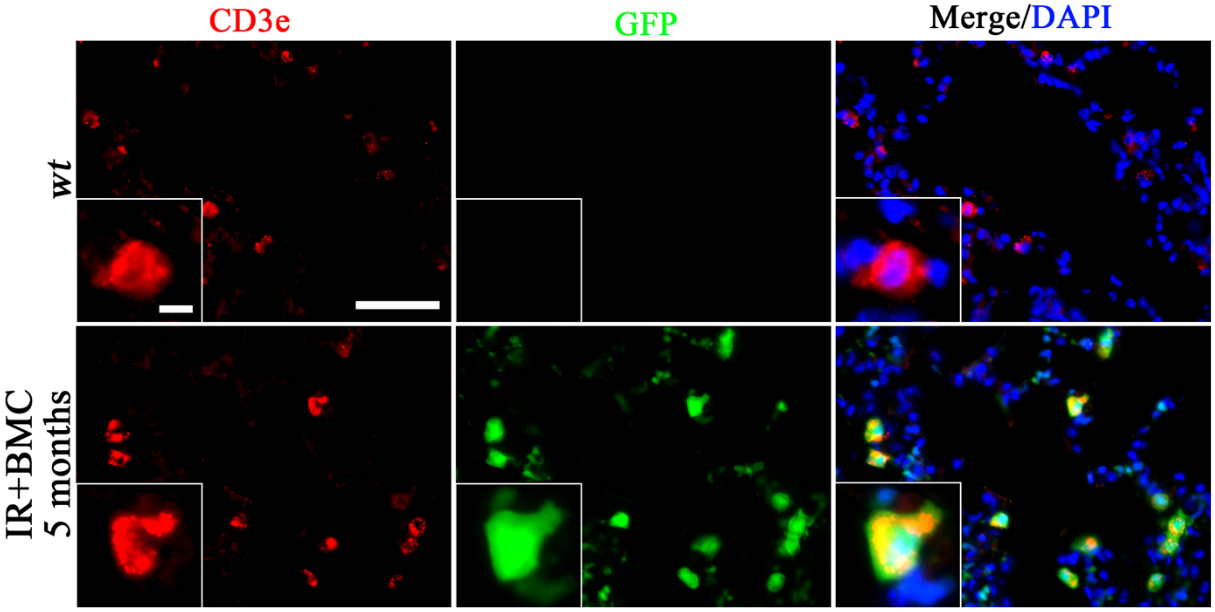

Figure E6

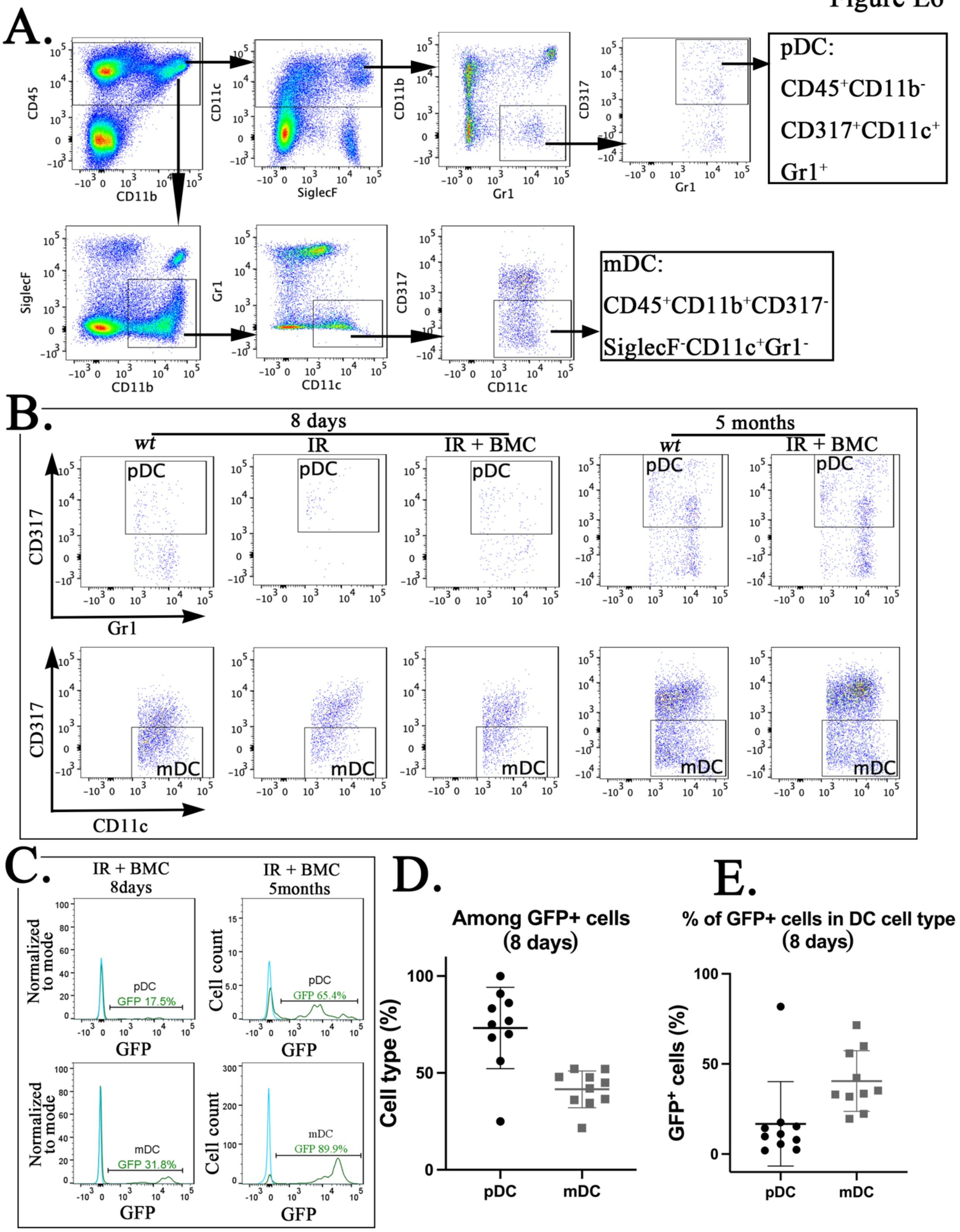
A.
pDC:
CD45⁺CD11b⁻
CD317⁺CD11c⁺
Gr1⁺
mDC:
CD45⁺CD11b⁺CD317⁻
SiglecF⁻CD11c⁺Gr1⁻
B.
8 days
5 months
wt
IR
IR + BMC
C.
IR + BMC
8days
IR + BMC
5months
pDC
GFP 17.5%
GFP 65.4%
mDC
GFP 31.8%
GFP 89.9%
D.
Among GFP+ cells
(8 days)
Cell type (%)
E.
% of GFP+ cells in DC cell type
(8 days)
GFP⁺ cells (%)

Figure E7

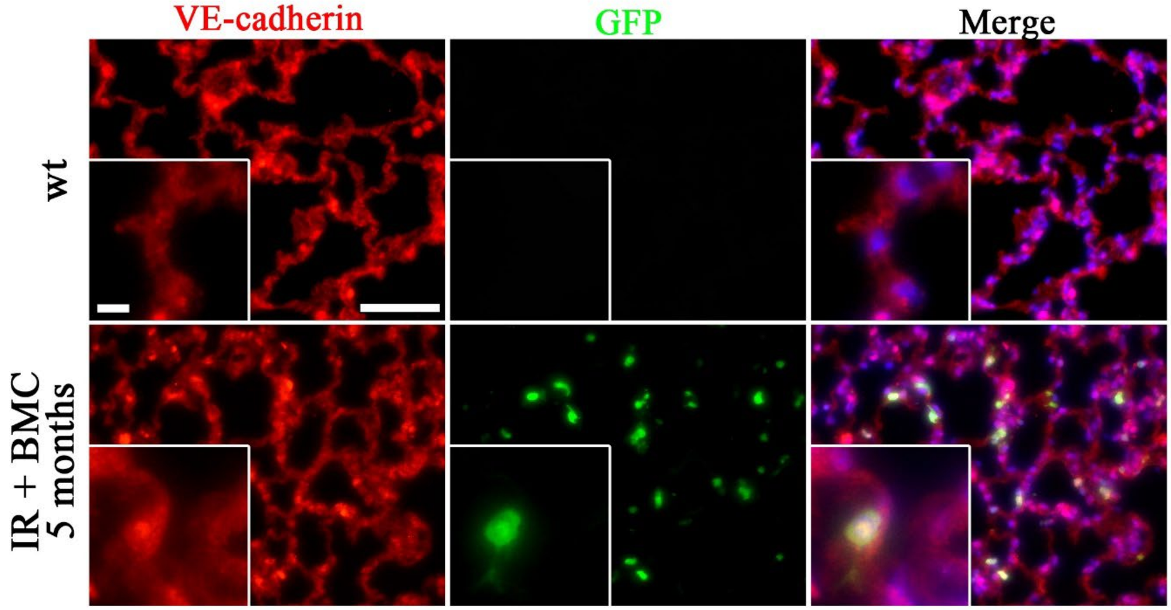

Figure E8

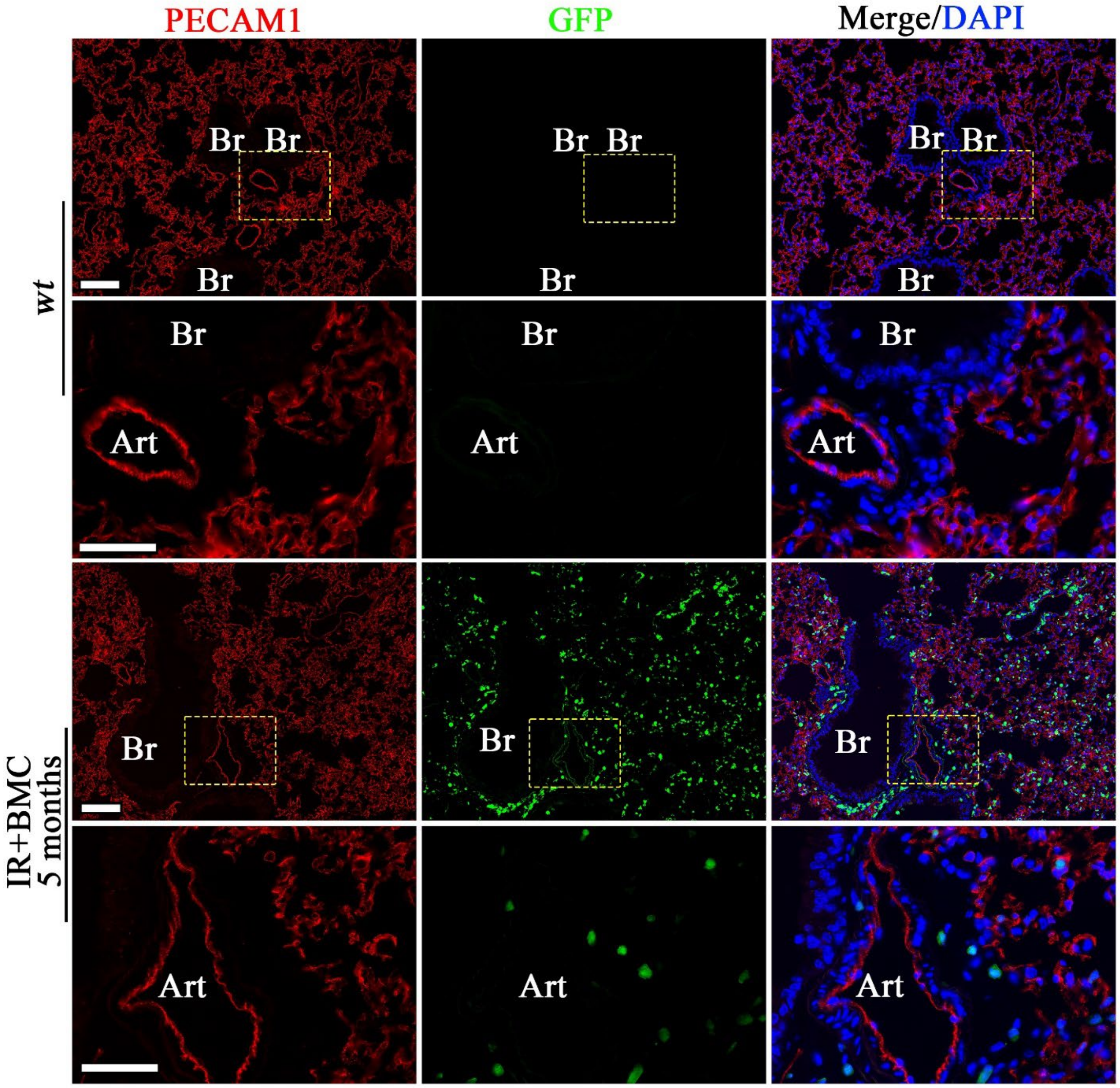

Figure E9

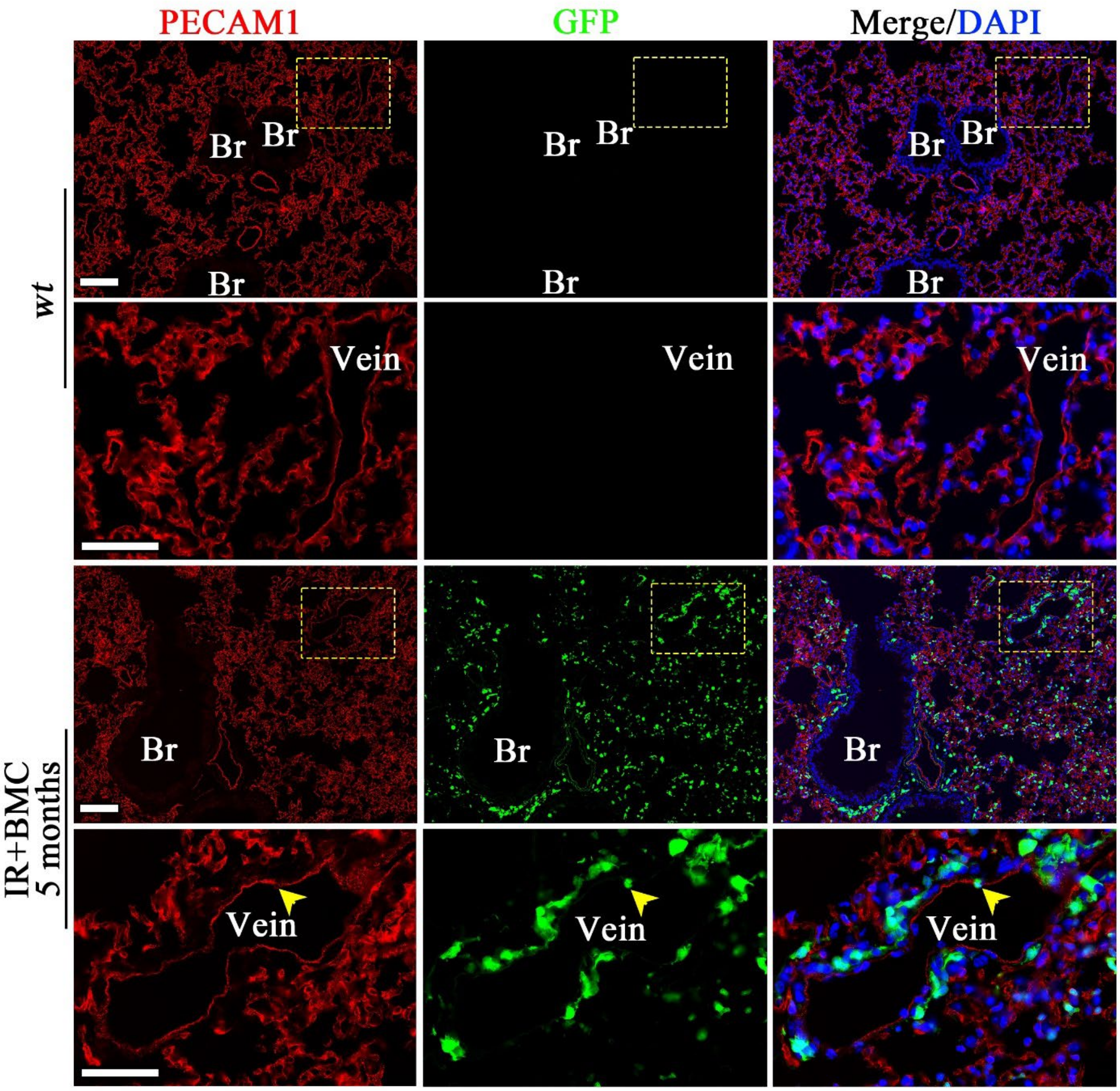

Figure E10

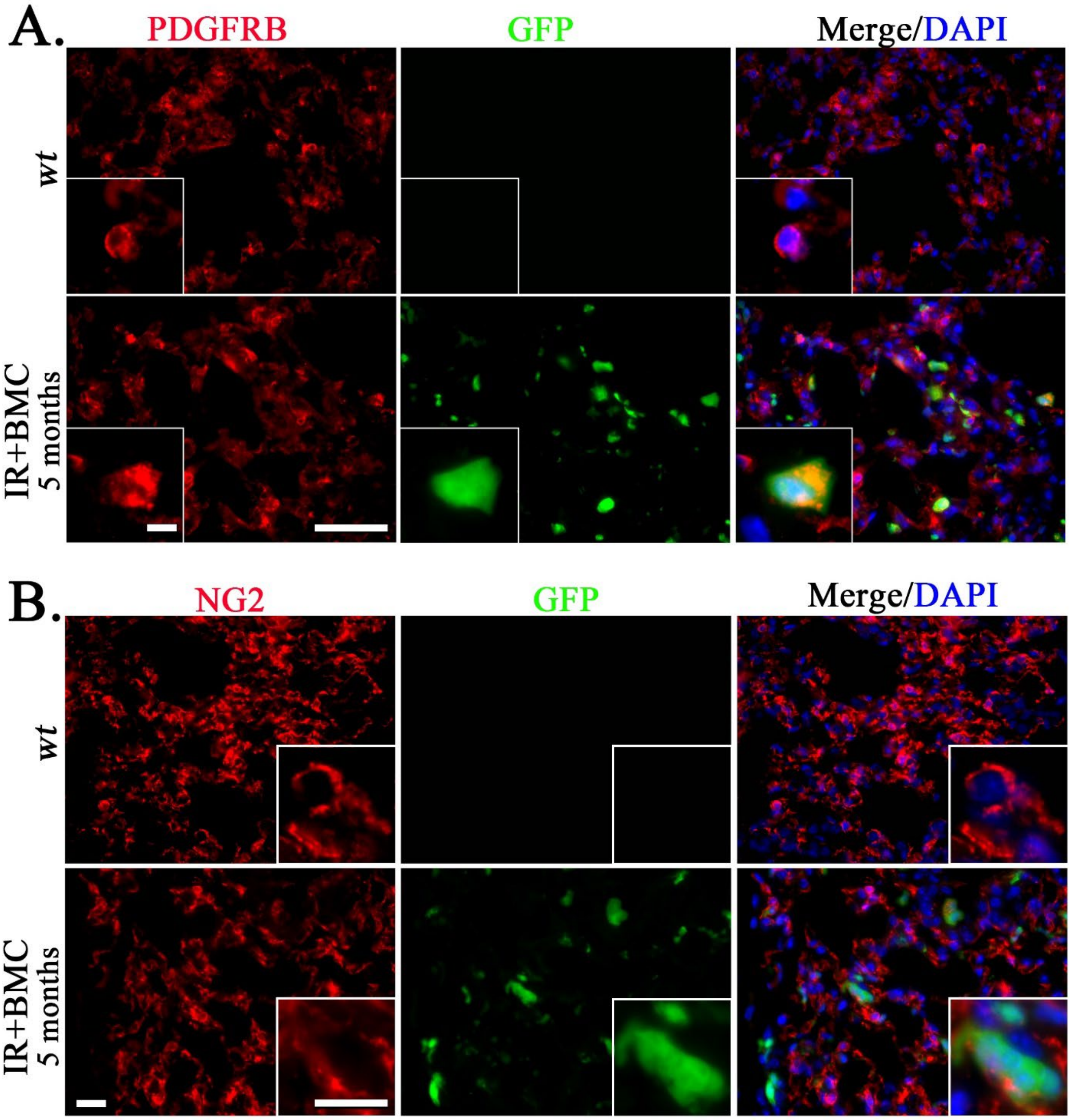
A.
PDGFRB
GFP
Merge/DAPI
wt
IR+BMC
5 months
B.
NG2
GFP
Merge/DAPI
wt
IR+BMC
5 months

Figure E11

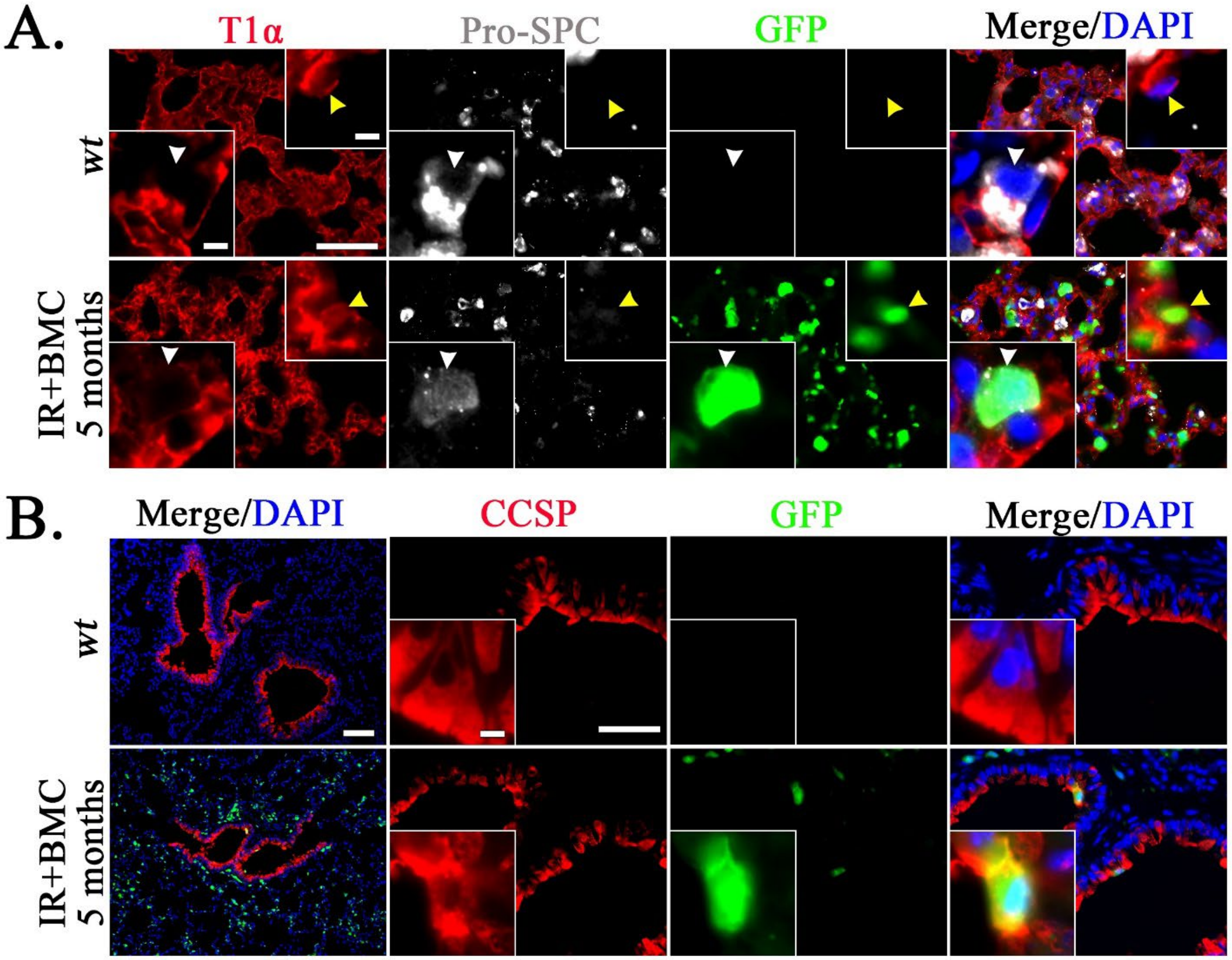
A.
T1α
Pro-SPC
GFP
Merge/DAPI
wt
IR+BMC
5 months
B.
Merge/DAPI
CCSP
GFP
Merge/DAPI
wt
IR+BMC
5 months

Figure E12

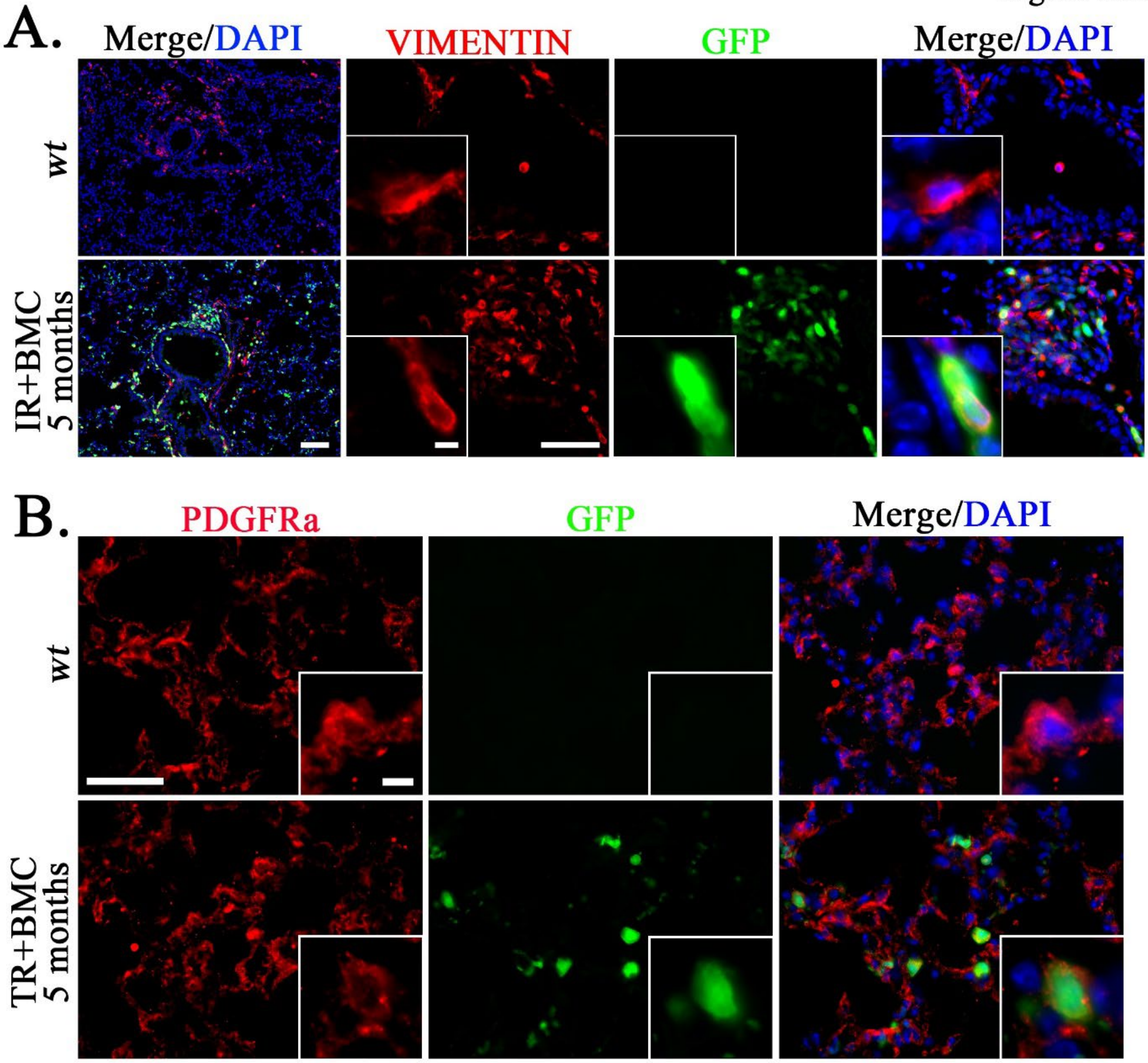
A.
Merge/DAPI
VIMENTIN
GFP
Merge/DAPI
wt
IR+BMC
5 months
B.
PDGFRa
GFP
Merge/DAPI
wt
TR+BMC
5 months

Figure E13

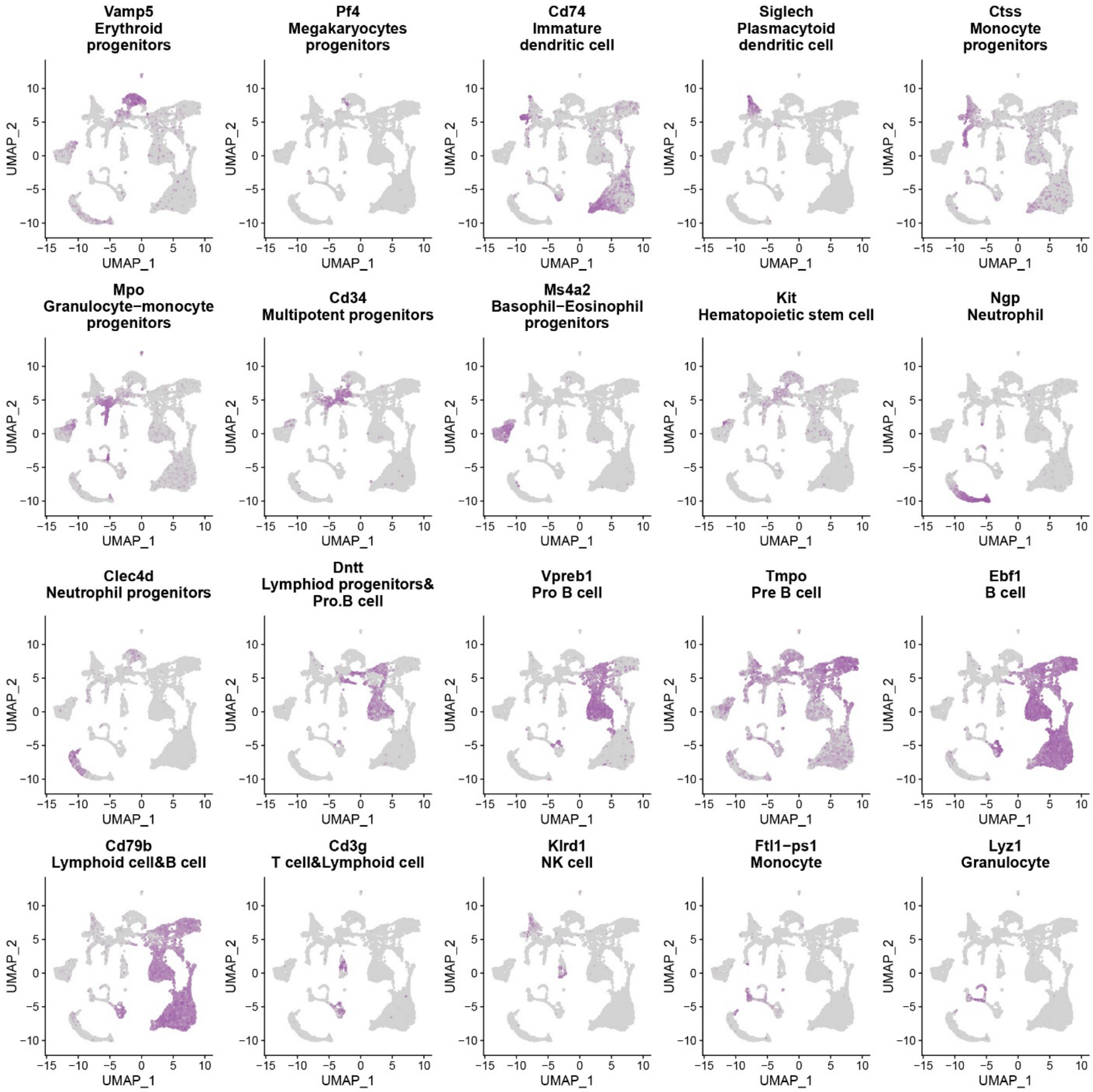
Vamp5
Erythroid progenitors
Pf4
Megakaryocytes progenitors
Cd74
Immature dendritic cell
Siglech
Plasmacytoid dendritic cell
Ctss
Monocyte progenitors
Mpo
Granulocyte−monocyte progenitors
Cd34
Multipotent progenitors
Ms4a2
Basophil−Eosinophil progenitors
Kit
Hematopoietic stem cell
Ngp
Neutrophil
Clec4d
Neutrophil progenitors
Dntt
Lymphiod progenitors&Pro.B cell
Vpreb1
Pro B cell
Tmpo
Pre B cell
Ebf1
B cell
Cd79b
Lymphoid cell&B cell
Cd3g
T cell&Lymphoid cell
Klrd1
NK cell
Ftl1−ps1
Monocyte
Lyz1
Granulocyte
UMAP_1
UMAP_2

Figure E14

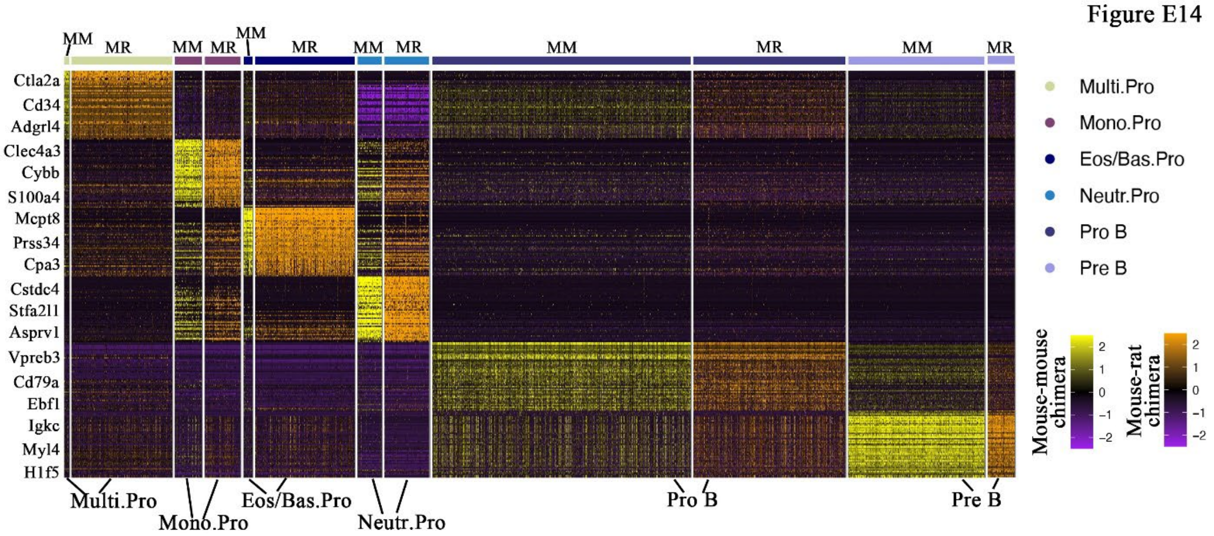
MM
MR
MM
MR
MM
MR
MM
MR
MM
MR
MM
MR
Ctla2a
Cd34
Adgrl4
Clec4a3
Cybb
S100a4
Mcpt8
Prss34
Cpa3
Cstdc4
Stfa2l1
Asprv1
Vpreb3
Cd79a
Ebf1
Igkc
Myl4
H1f5
Multi.Pro
Mono.Pro
Eos/Bas.Pro
Neutr.Pro
Pro B
Pre B
Mouse-mouse chimera
Mouse-rat chimera
2
1
0
−1
−2

Figure E15

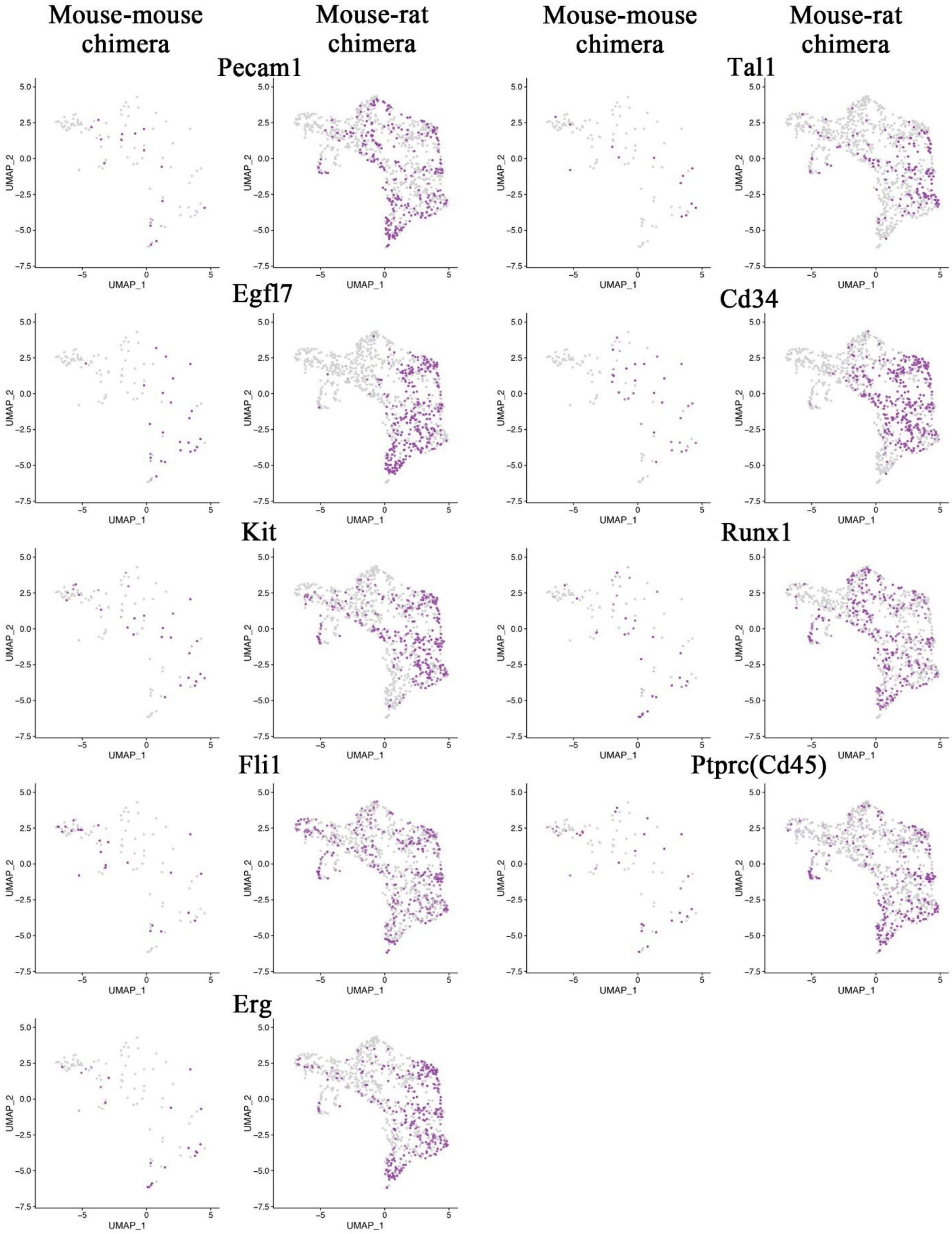
Mouse-mouse chimera
Mouse-rat chimera
Mouse-mouse chimera
Mouse-rat chimera
Pecam1
Tal1
Egfl7
Cd34
Kit
Runx1
Fli1
Ptprc(Cd45)
Erg
UMAP_1
UMAP_2

Figure E16

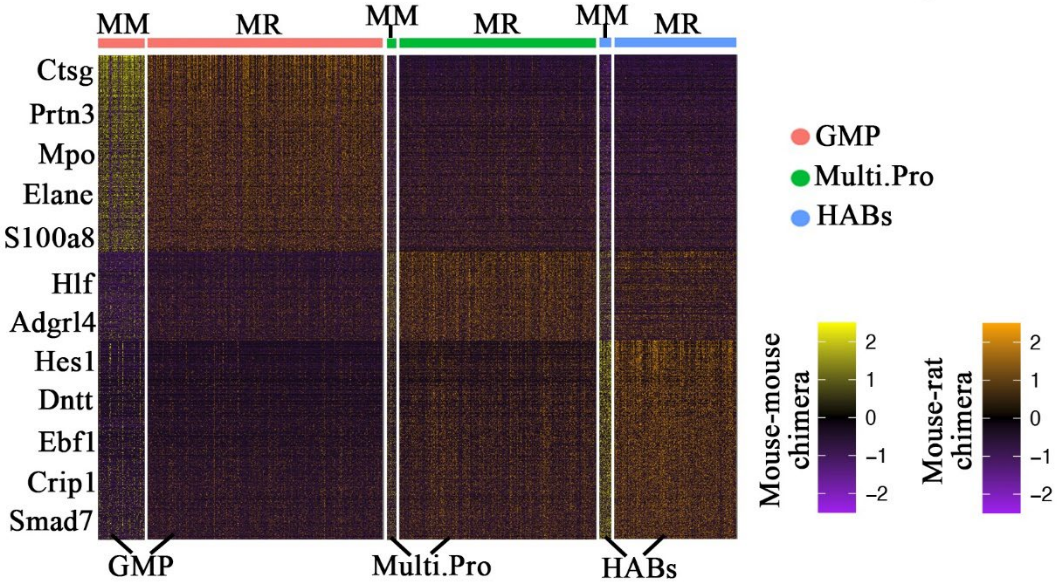
MM
MR
MM
MR
MM
MR
Ctsg
Prtn3
Mpo
Elane
S100a8
Hlf
Adgrl4
Hes1
Dntt
Ebf1
Crip1
Smad7
GMP
Multi.Pro
HABs
GMP
Multi.Pro
HABs
Mouse-mouse chimera
2
1
0
−1
−2
Mouse-rat chimera
2
1
0
−1
−2

Figure E17

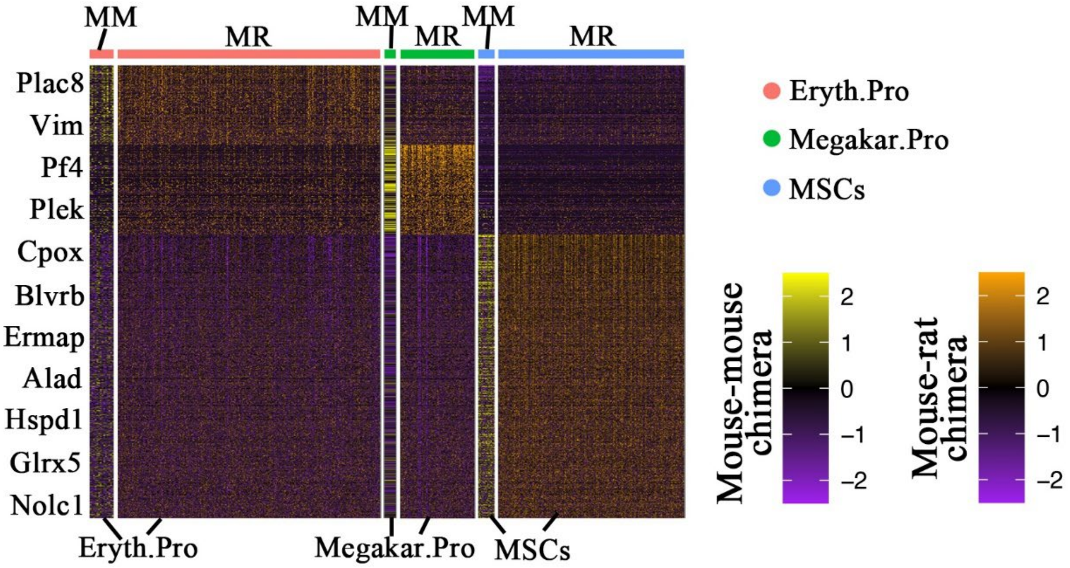
MM
MR
MM
MR
MM
MR
Plac8
Vim
Pf4
Plek
Cpox
Blvrb
Ermap
Alad
Hspd1
Glrx5
Nolc1
Eryth.Pro
Megakar.Pro
MSCs
Eryth.Pro
Megakar.Pro
MSCs
Mouse-mouse chimera
2
1
0
−1
−2
Mouse-rat chimera
2
1
0
−1
−2

Figure E18

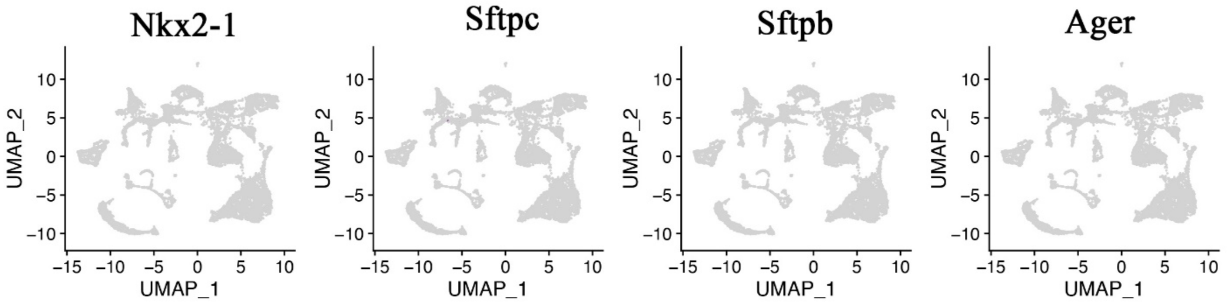

Supplement: Supplementary file 1 [file DataSheet1.PDF]
